# Supplementary figures and images for: Reserpine maintains photoreceptor survival in retinal ciliopathy by resolving proteostasis imbalance and ciliogenesis defects
Source: eLife. 2023 Mar 28;12:e83205. doi: 10.7554/eLife.83205 (PMC10121224; doi:10.7554/eLife.83205)

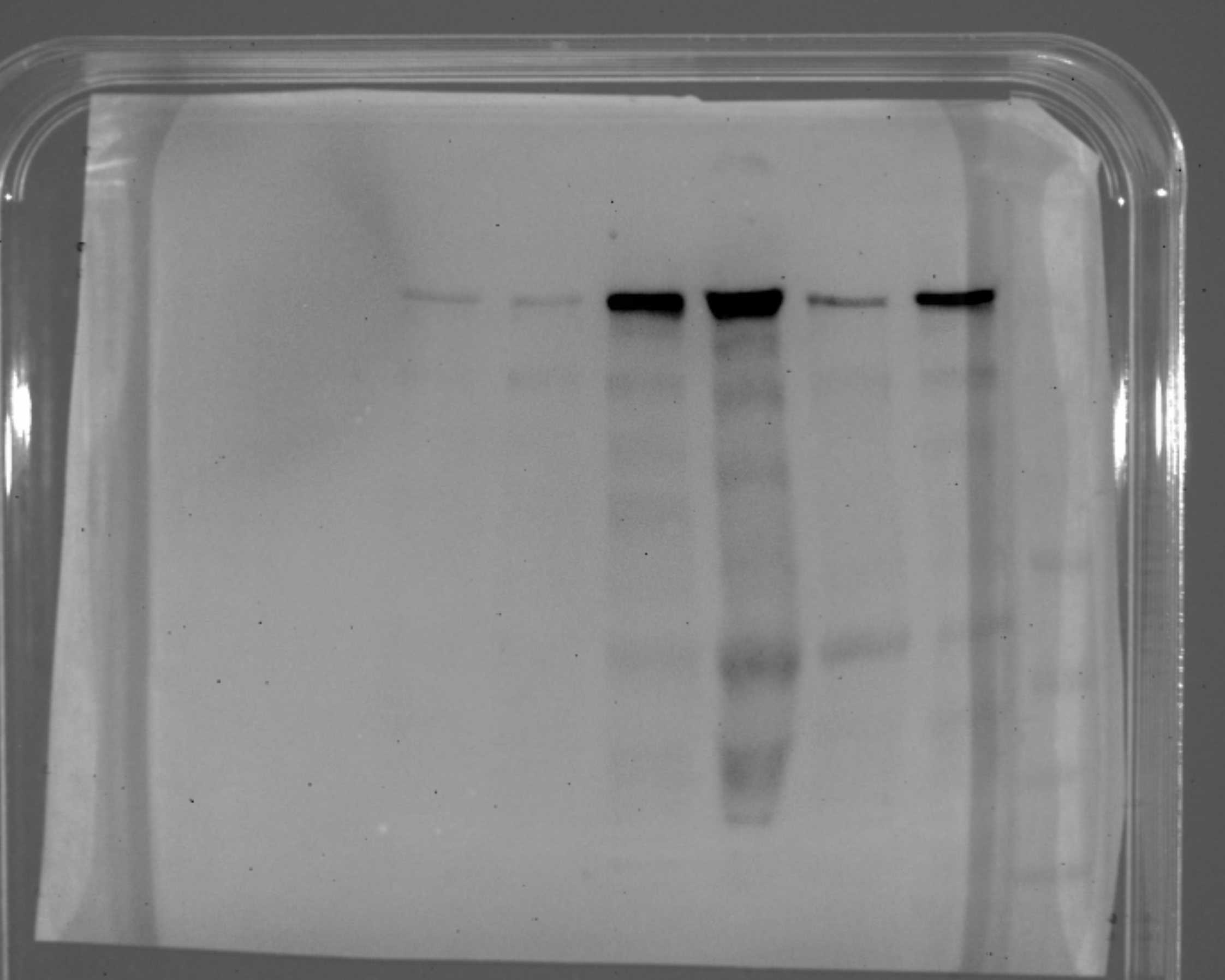

Supplement: Figure 3—figure supplement 1—source data 1. — The size of the protein ladders, CEP290, and relevant sample identity are labeled. [file elife-83205-fig3-figsupp1-data1.zip › Figure 3ΓÇöfigure supplement 1-source data 1/Figure 3-figure supplement 1-source data 1_Composite.tif]

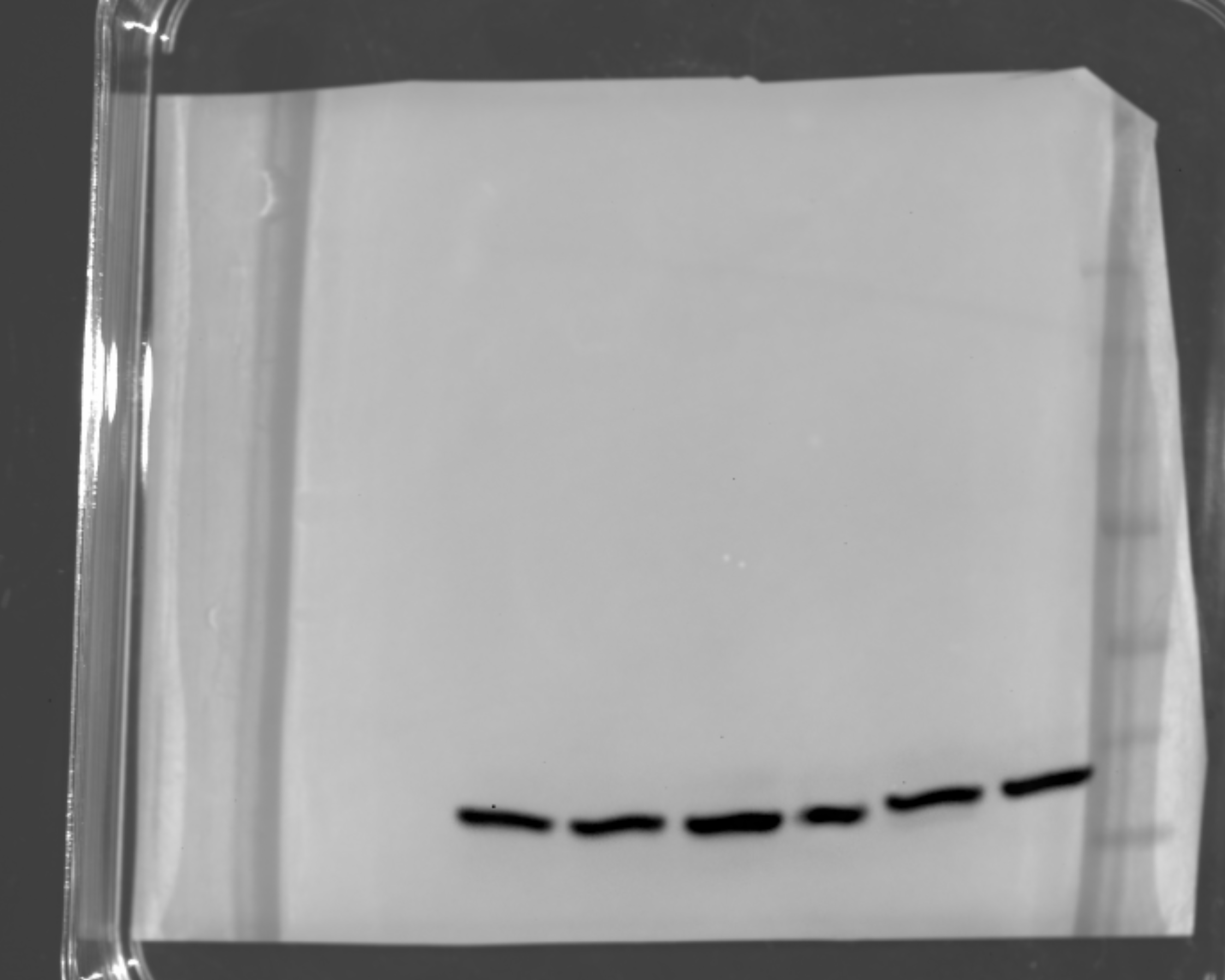

Supplement: Figure 3—figure supplement 1—source data 2. — The size of the protein ladders, GAPGH, and relevant sample identity are labeled. [file elife-83205-fig3-figsupp1-data2.zip › Figure 3ΓÇöfigure supplement 1-source data 2/Figure 3ΓÇöfigure supplement 1-source data 2_Composite.tif]

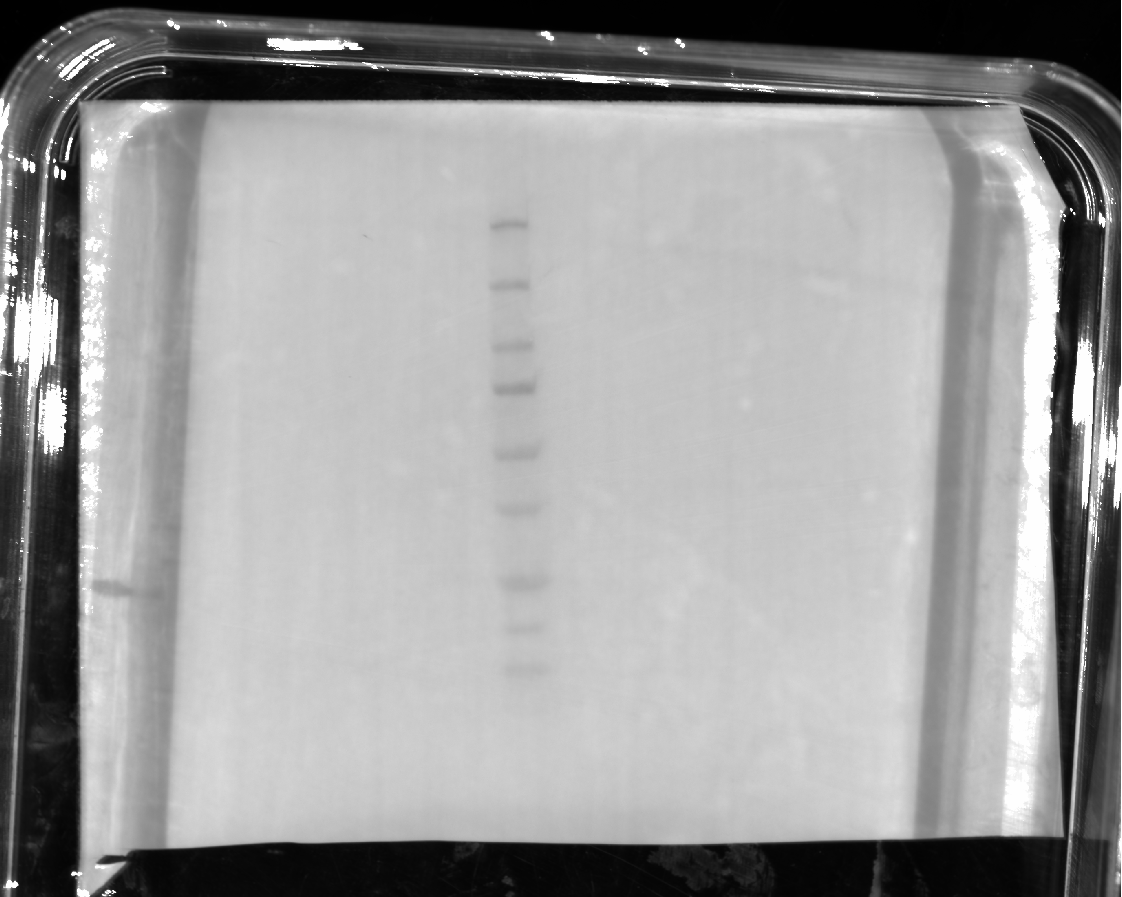

Supplement: Figure 5—source data 1. — RSP stands for reserpine. The size of the protein ladders, p62, and relevant sample identity are labeled. [file elife-83205-fig5-data1.zip › Figure 5-source data 1/Figure 5-source data 1_Colorimetric.tif]

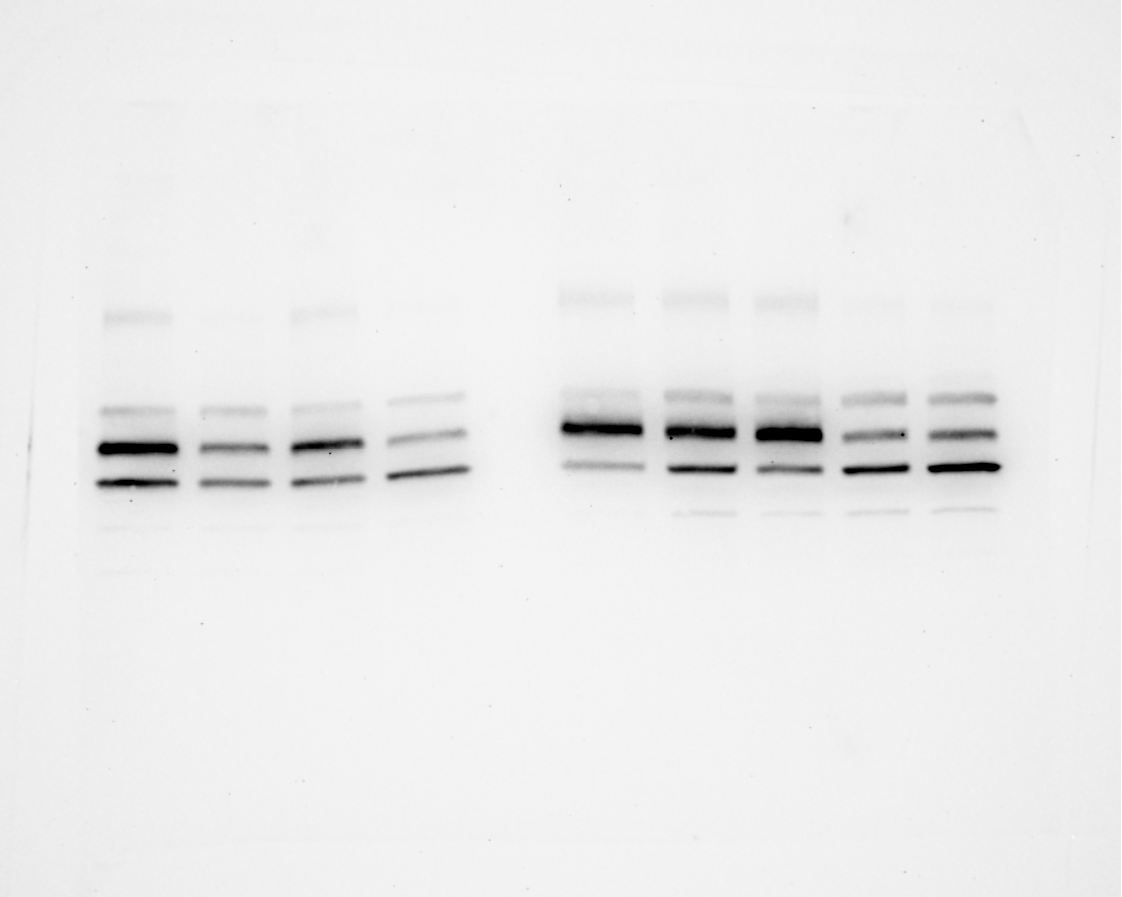

Supplement: Figure 5—source data 1. — RSP stands for reserpine. The size of the protein ladders, p62, and relevant sample identity are labeled. [file elife-83205-fig5-data1.zip › Figure 5-source data 1/Figure 5-source data 1_Chemiluminescence.tif]

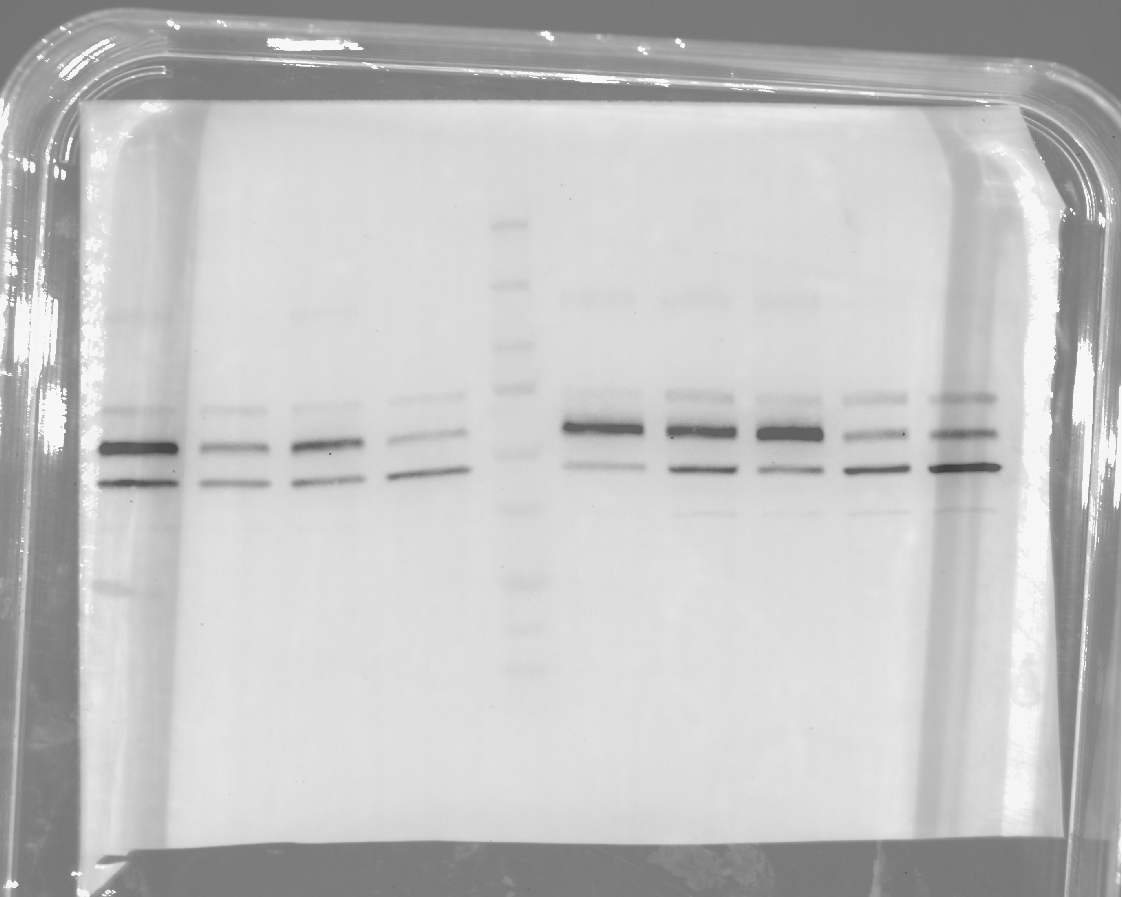

Supplement: Figure 5—source data 1. — RSP stands for reserpine. The size of the protein ladders, p62, and relevant sample identity are labeled. [file elife-83205-fig5-data1.zip › Figure 5-source data 1/Figure 5-source data 1_Composite.tif]

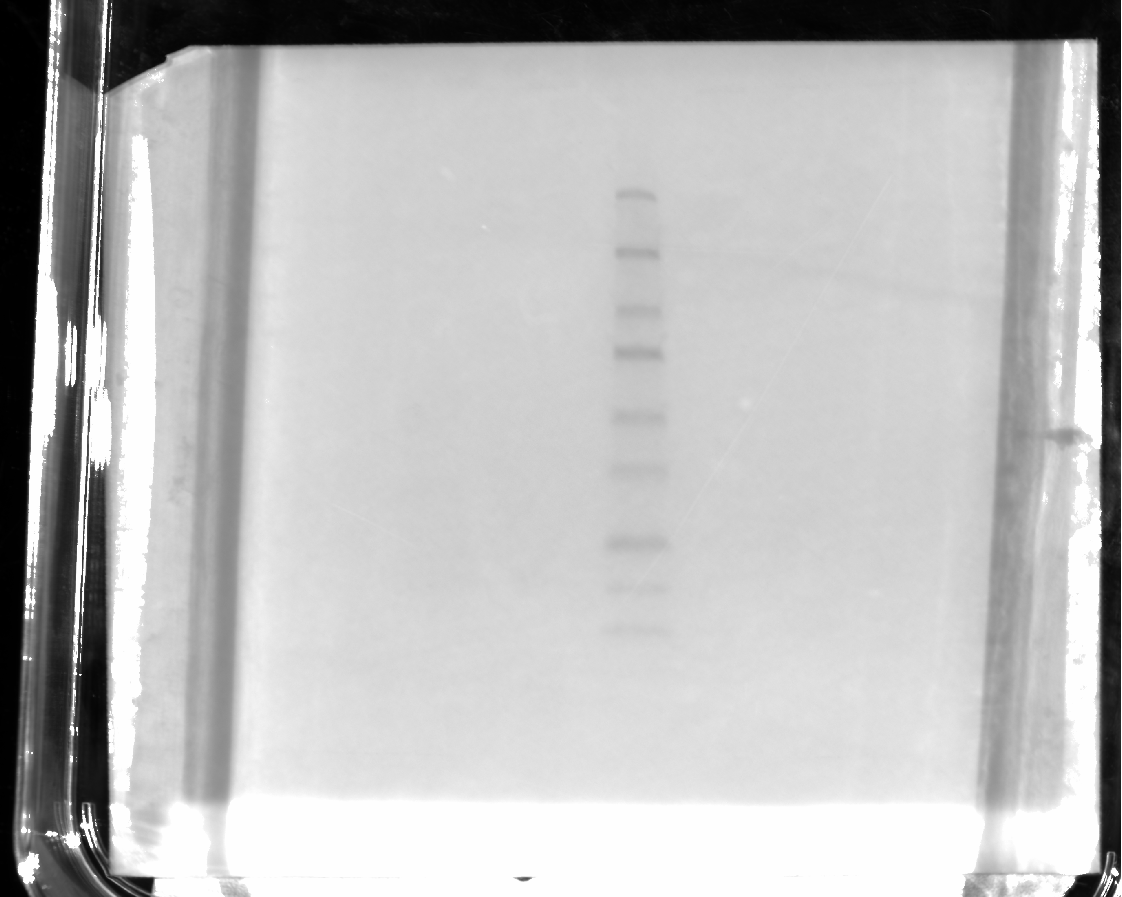

Supplement: Figure 5—source data 2. — RSP stands for reserpine. The size of the protein ladders, LC3, and relevant sample identity are labeled. [file elife-83205-fig5-data2.zip › Figure 5-source data 2/Figure 5-source data 2_Colorimetric.tif]

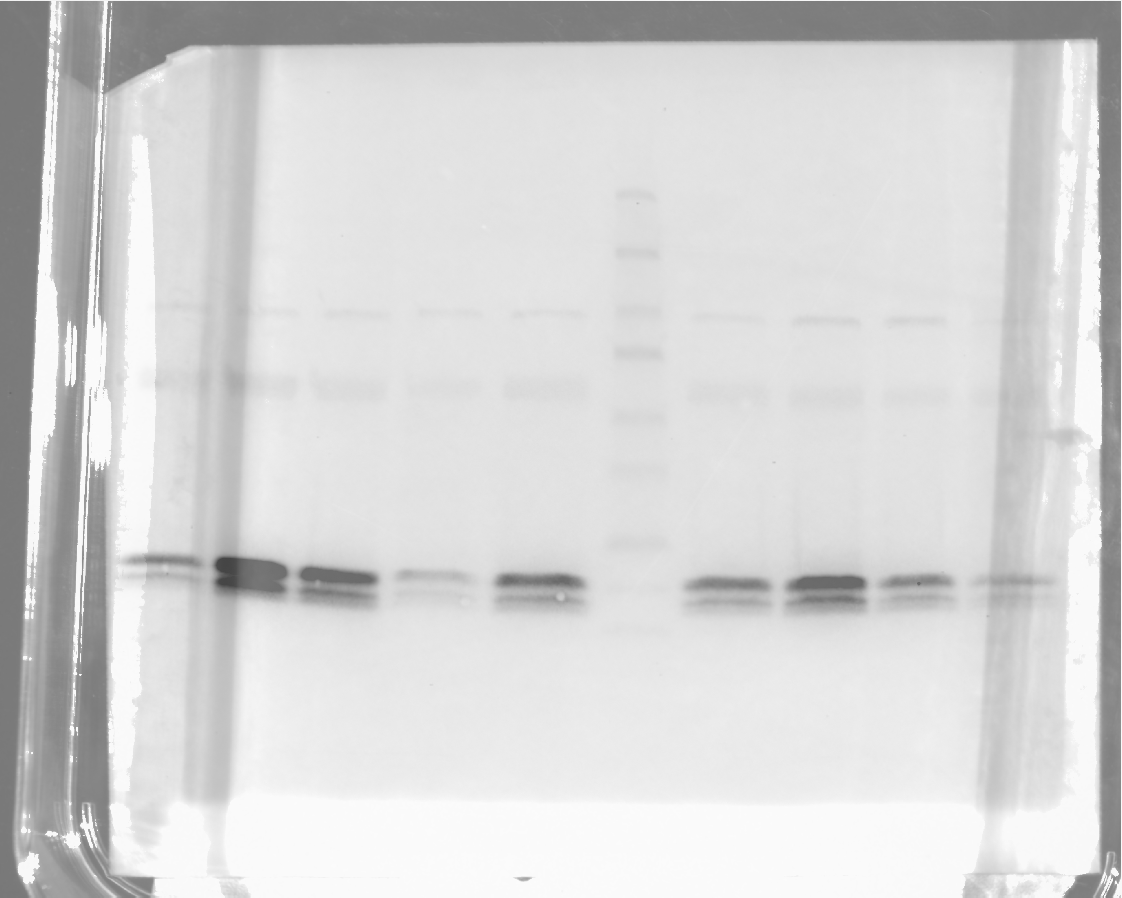

Supplement: Figure 5—source data 2. — RSP stands for reserpine. The size of the protein ladders, LC3, and relevant sample identity are labeled. [file elife-83205-fig5-data2.zip › Figure 5-source data 2/Figure 5-source data 2_Composite.tif]

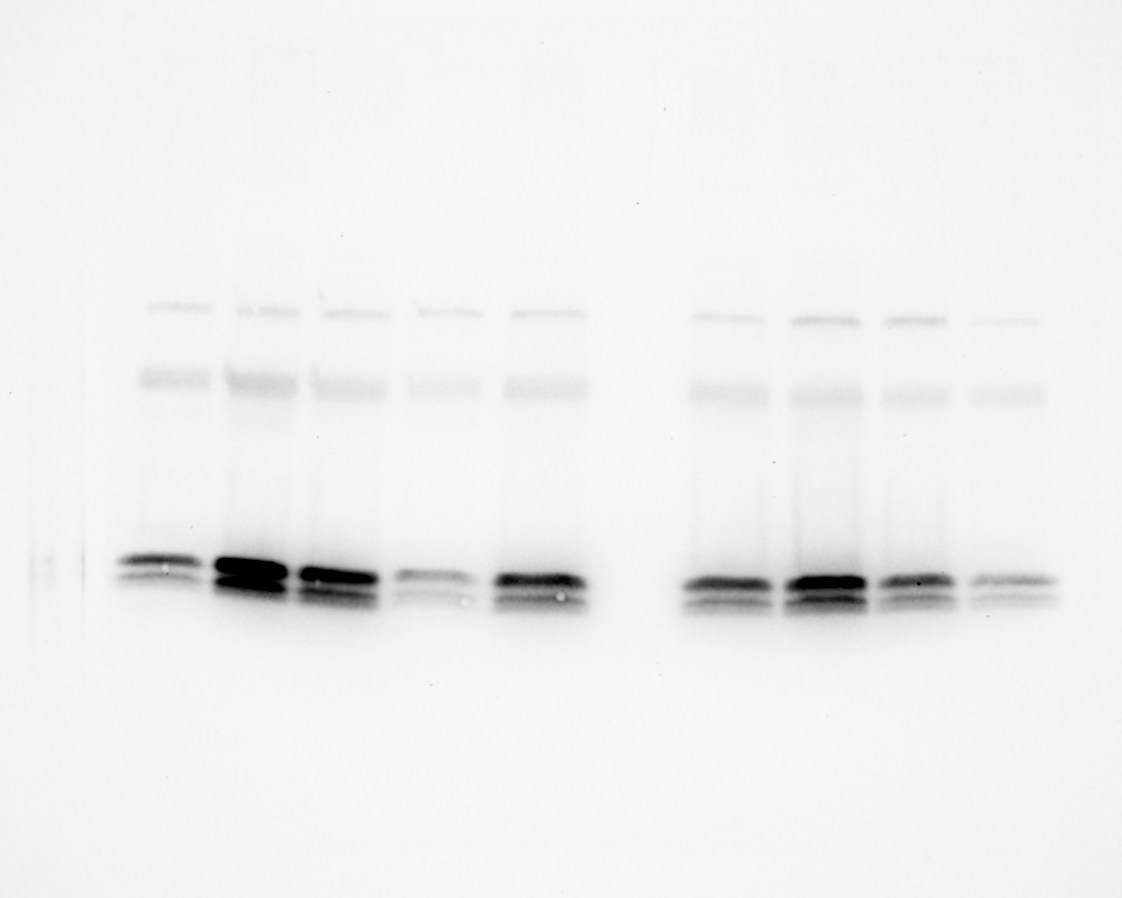

Supplement: Figure 5—source data 2. — RSP stands for reserpine. The size of the protein ladders, LC3, and relevant sample identity are labeled. [file elife-83205-fig5-data2.zip › Figure 5-source data 2/Figure 5-source data 2_Chemiluminescence.tif]

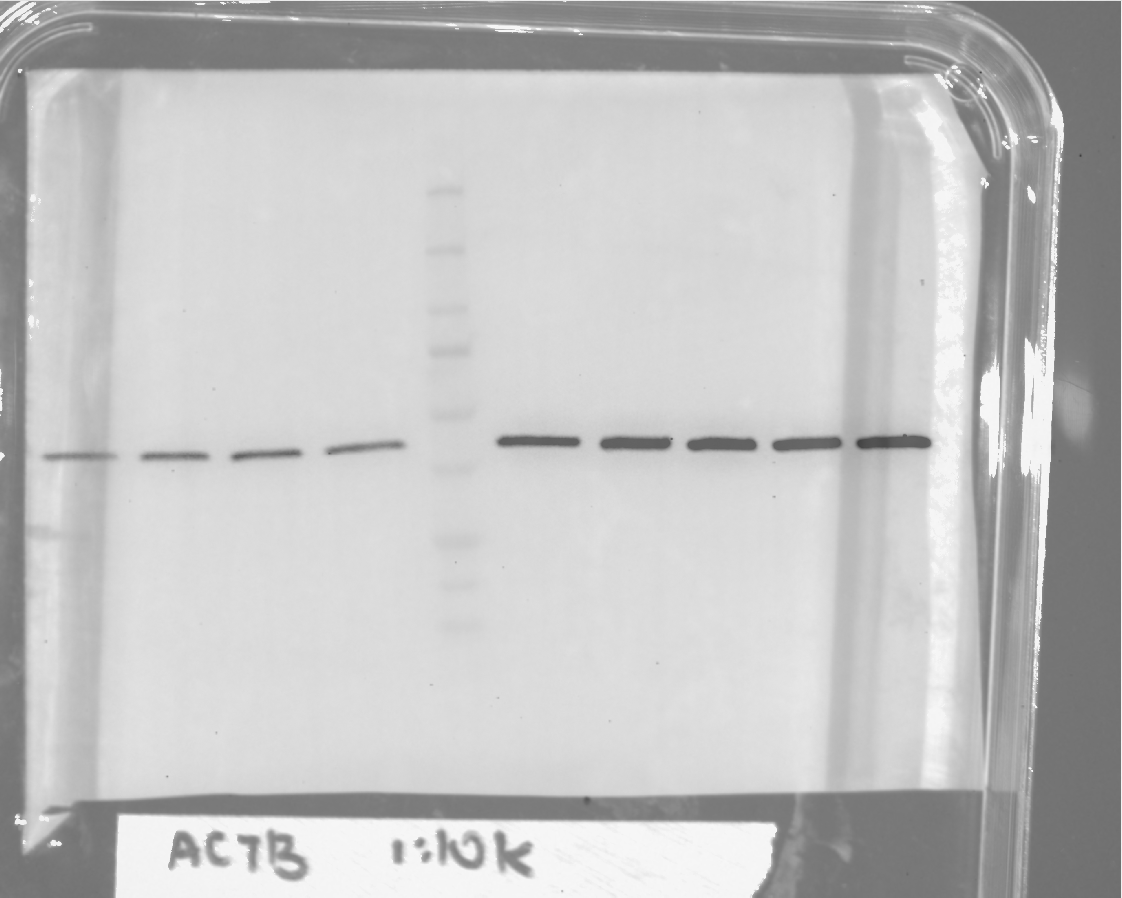

Supplement: Figure 5—source data 3. — RSP stands for reserpine. The size of the protein ladders, β-Actin, and relevant sample identity are labeled. [file elife-83205-fig5-data3.zip › Figure 5-source data 3/Figure 5-source data 3_Composite.tif]

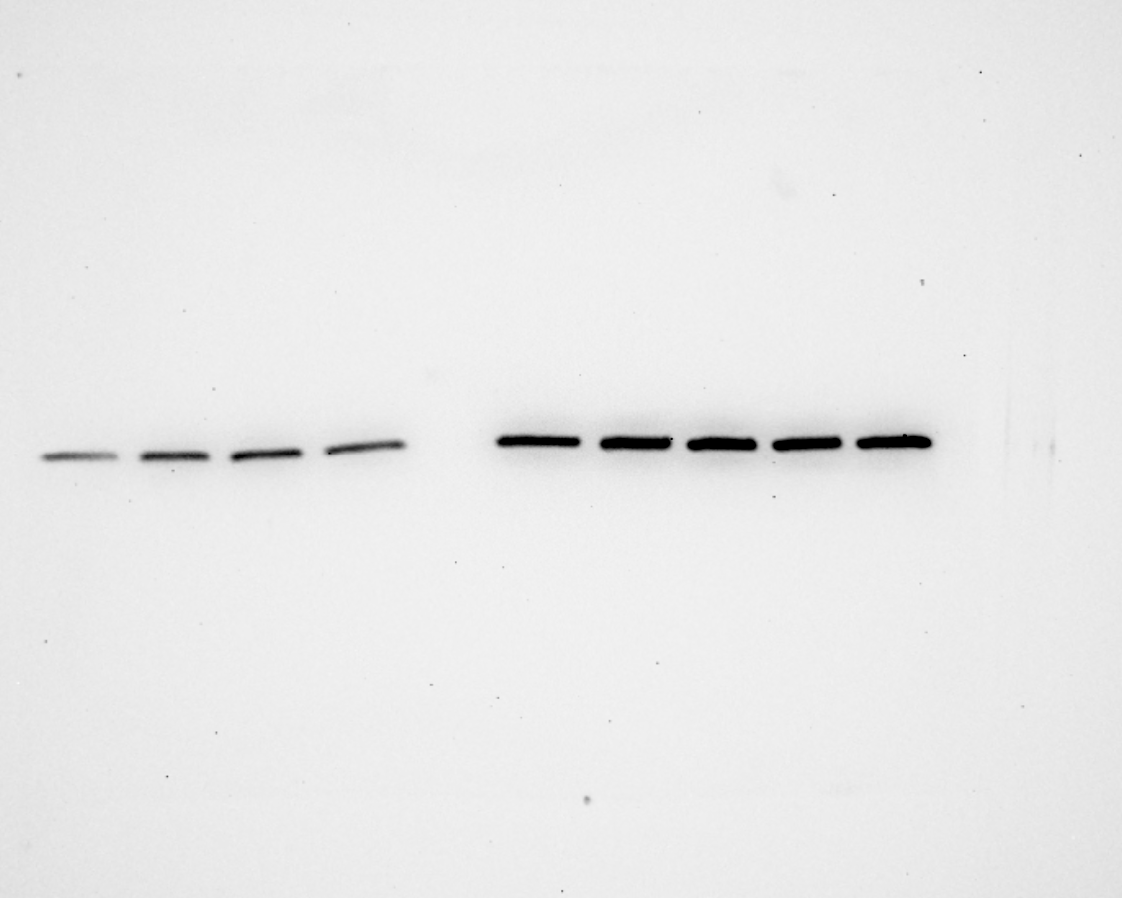

Supplement: Figure 5—source data 3. — RSP stands for reserpine. The size of the protein ladders, β-Actin, and relevant sample identity are labeled. [file elife-83205-fig5-data3.zip › Figure 5-source data 3/Figure 5-source data 3_Chemiluminescence.tif]

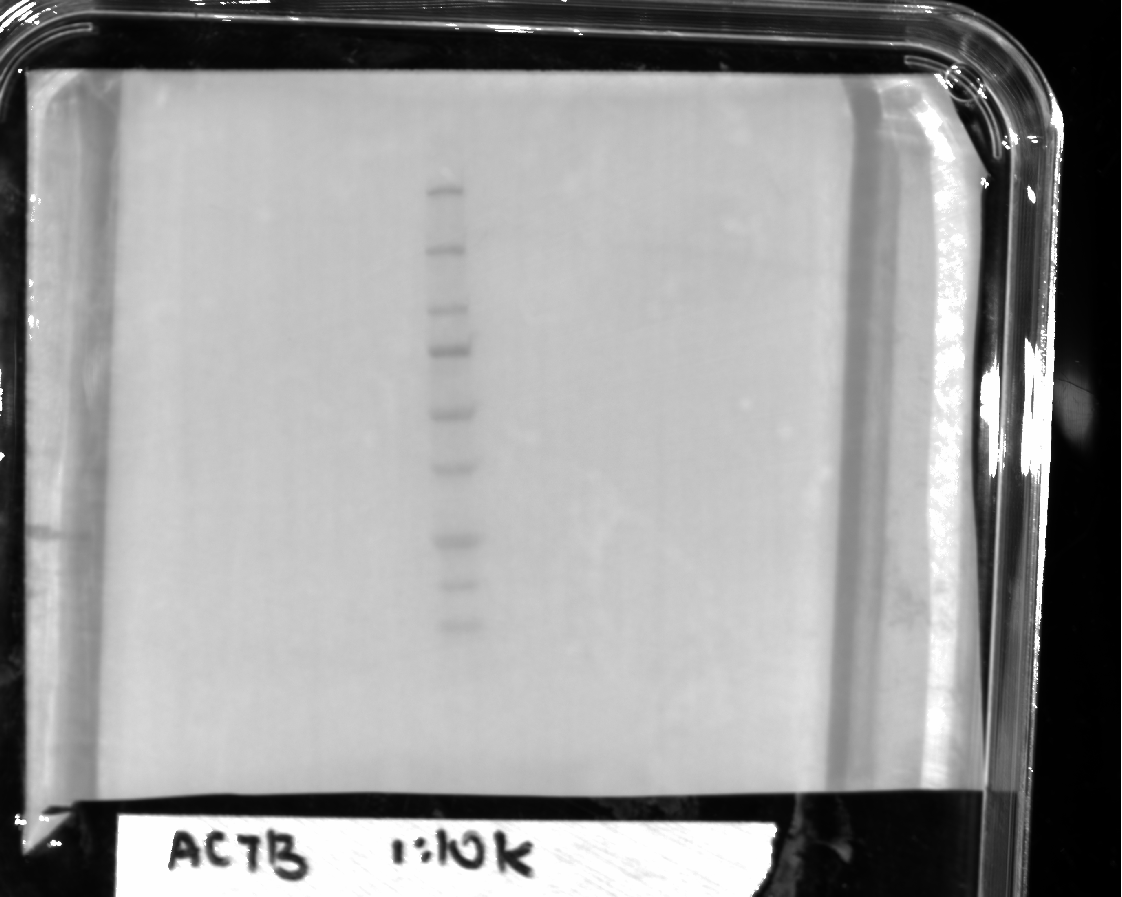

Supplement: Figure 5—source data 3. — RSP stands for reserpine. The size of the protein ladders, β-Actin, and relevant sample identity are labeled. [file elife-83205-fig5-data3.zip › Figure 5-source data 3/Figure 5-source data 3_Colorimetric.tif]

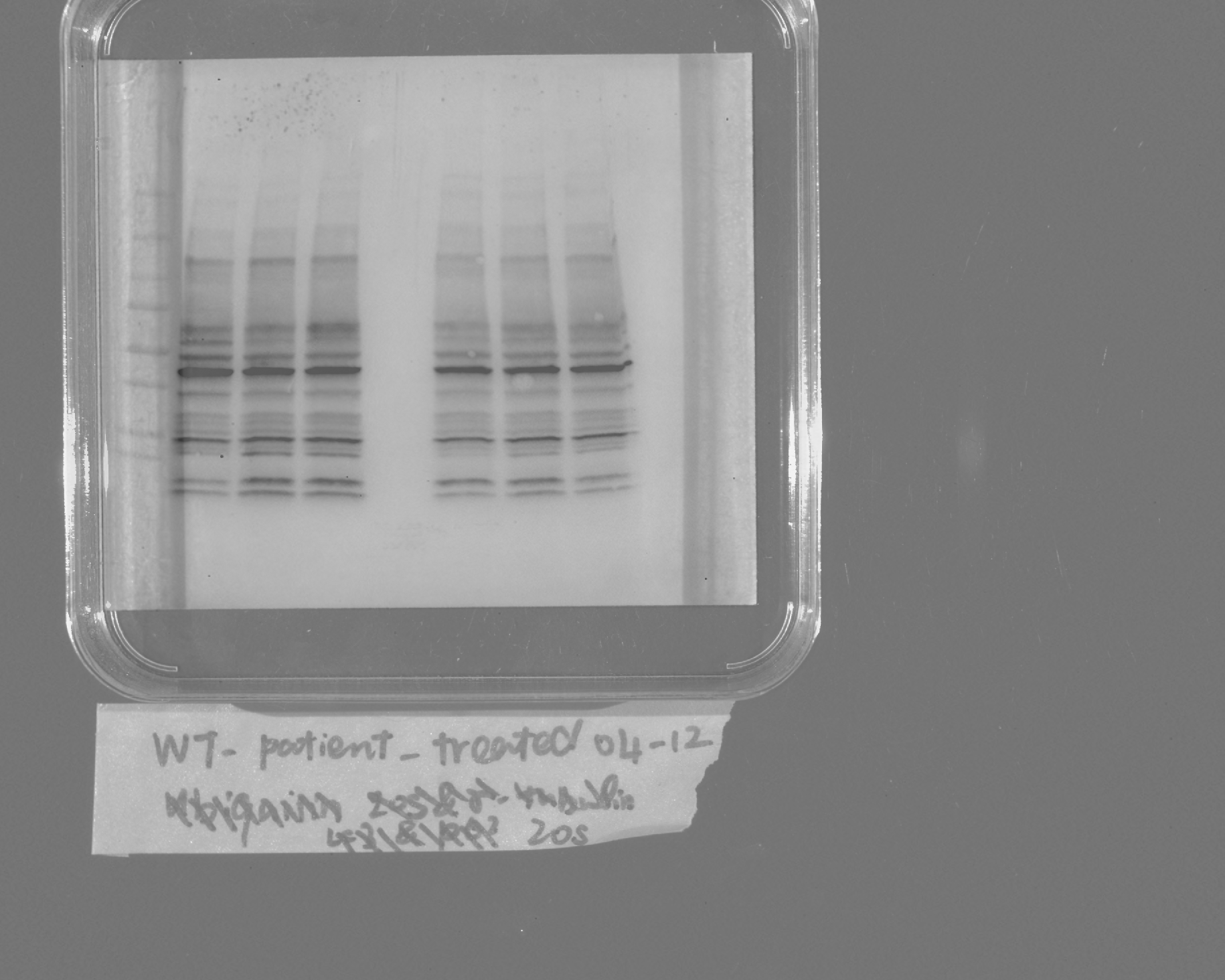

Supplement: Figure 5—source data 4. — RSP stands for reserpine. The size of the protein ladders, β-Actin, 20S proteosome, and relevant sample identity are labeled. [file elife-83205-fig5-data4.zip › Figure 5-source data 4/Figure 5-source data 4_Composite.tif]

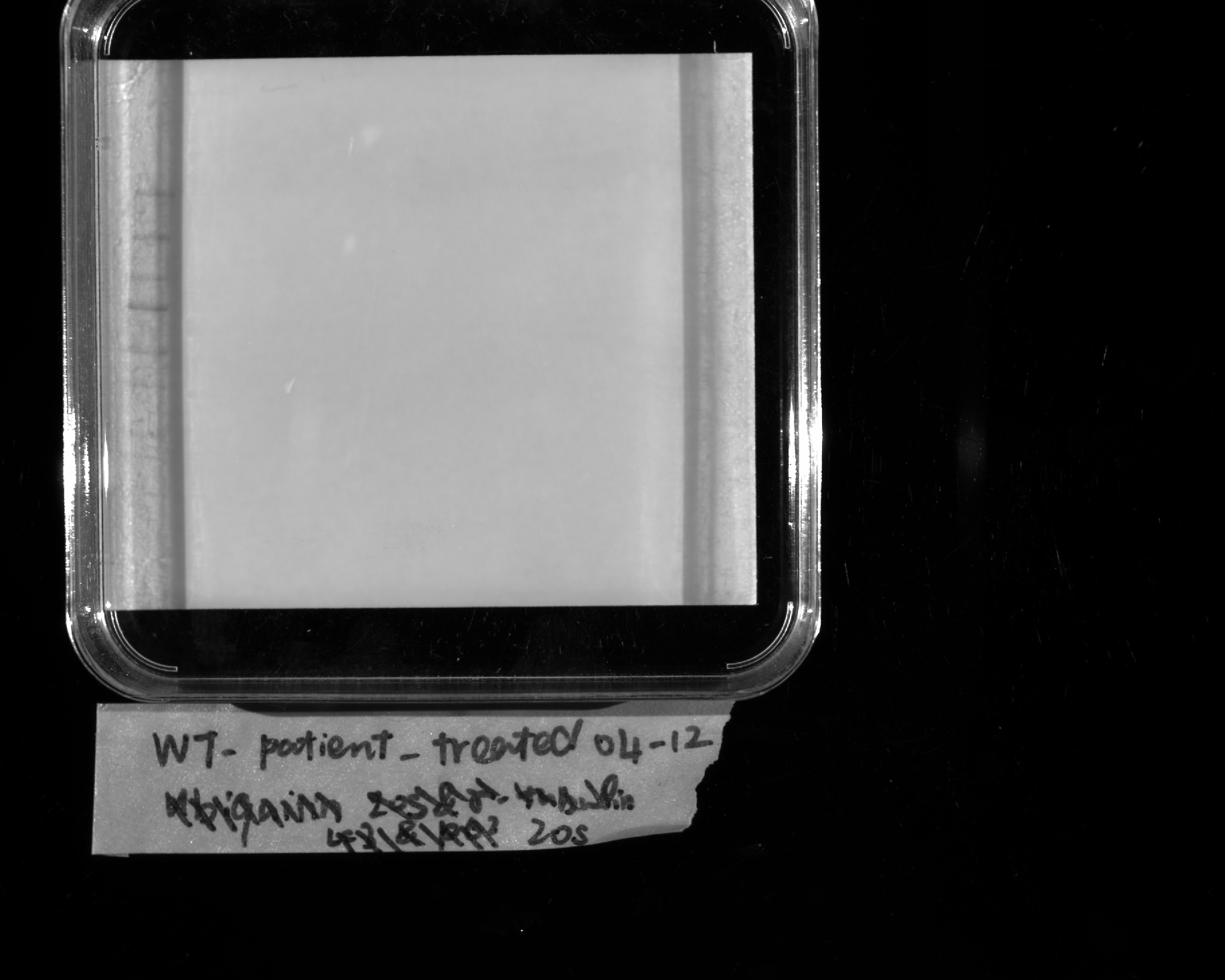

Supplement: Figure 5—source data 4. — RSP stands for reserpine. The size of the protein ladders, β-Actin, 20S proteosome, and relevant sample identity are labeled. [file elife-83205-fig5-data4.zip › Figure 5-source data 4/Figure 5-source data 4_Colorimetric.tif]

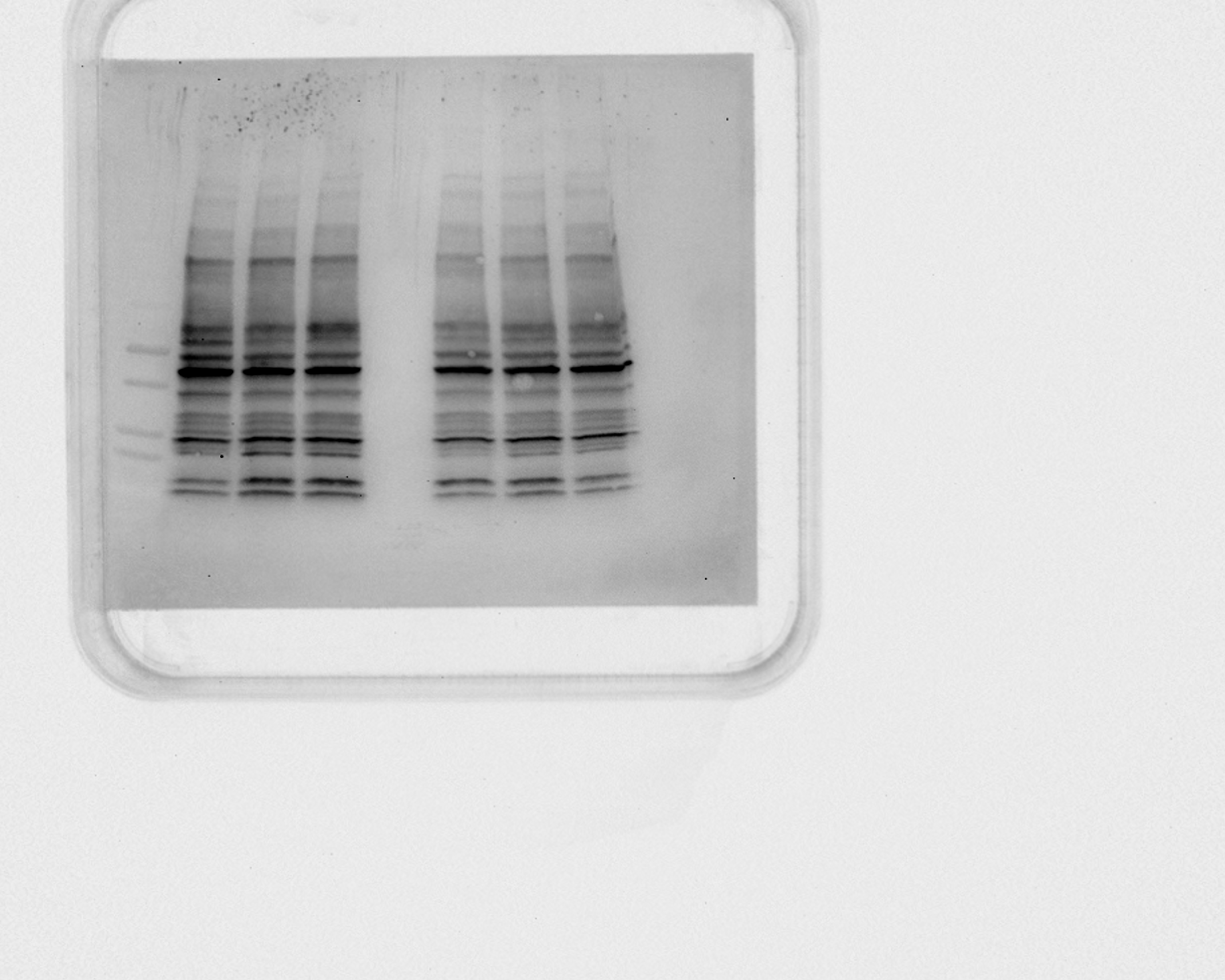

Supplement: Figure 5—source data 4. — RSP stands for reserpine. The size of the protein ladders, β-Actin, 20S proteosome, and relevant sample identity are labeled. [file elife-83205-fig5-data4.zip › Figure 5-source data 4/Figure 5-source data 4_Chemiluminescence.tif]

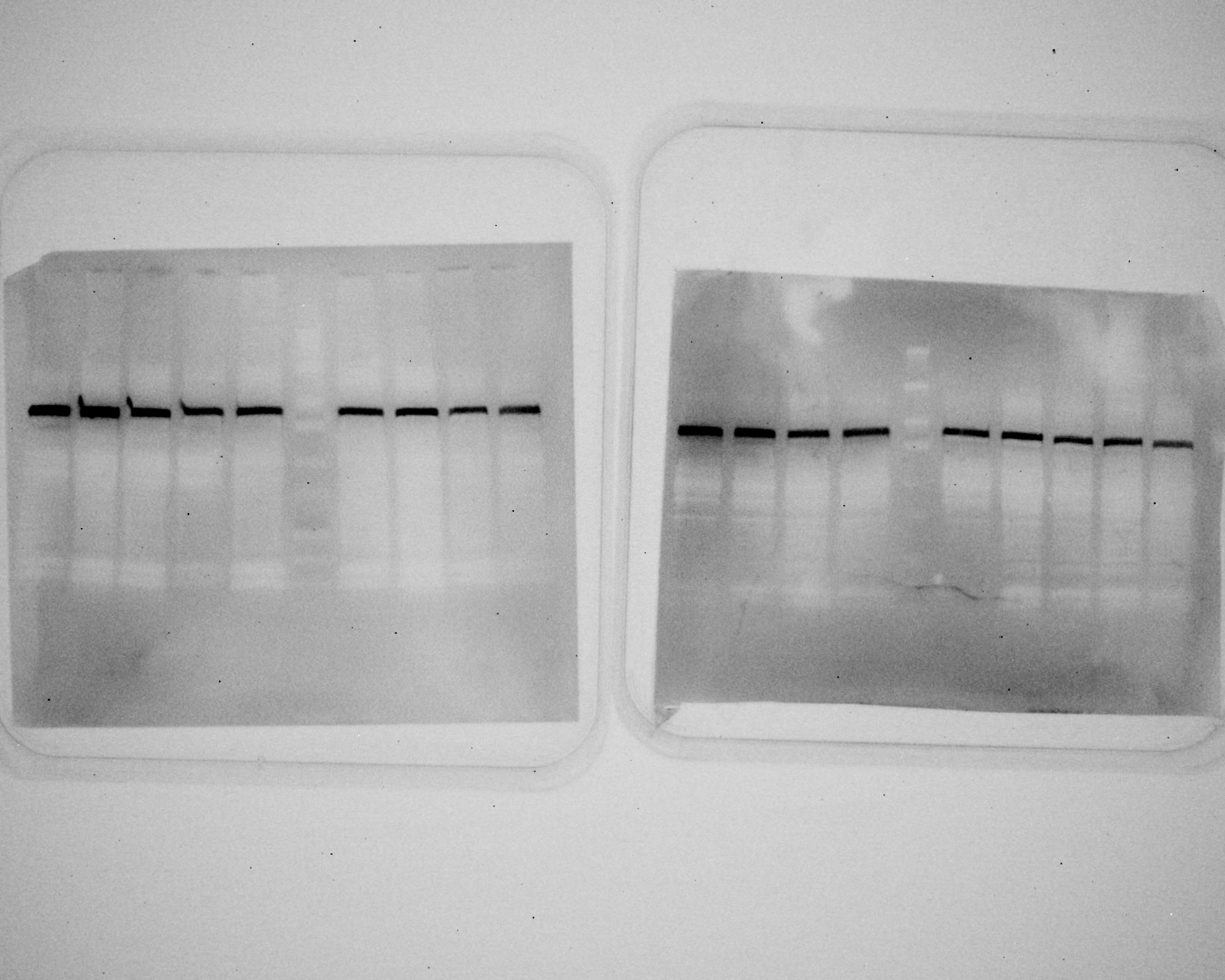

Supplement: Figure 5—source data 5. — RSP stands for reserpine. The size of the protein ladders, IFT88, and relevant sample identity are labeled. [file elife-83205-fig5-data5.zip › Figure 5-source data 5/Figure 5-source data 5_Chemiluminescence.tif]

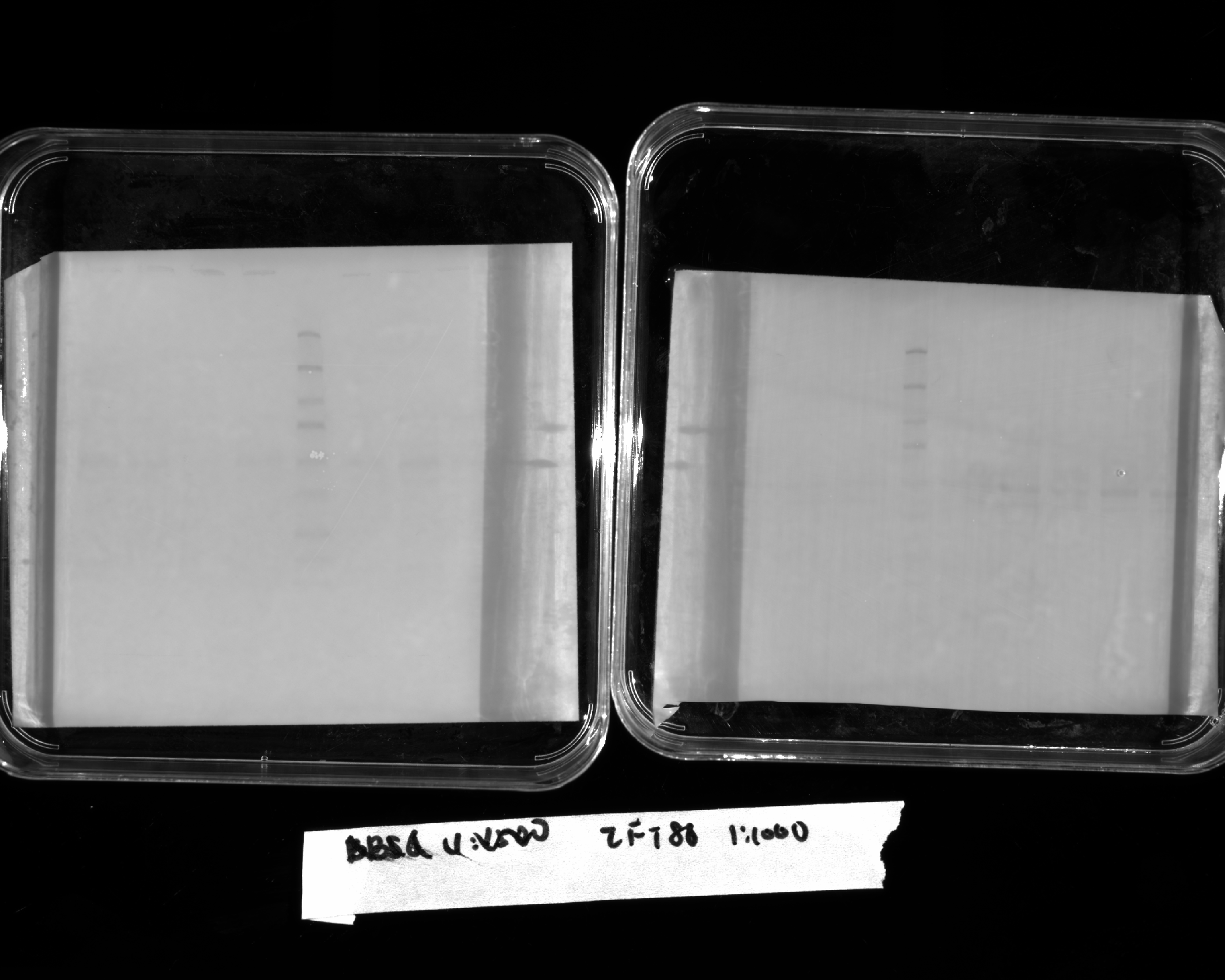

Supplement: Figure 5—source data 5. — RSP stands for reserpine. The size of the protein ladders, IFT88, and relevant sample identity are labeled. [file elife-83205-fig5-data5.zip › Figure 5-source data 5/Figure 5-source data 5_Colorimetric.tif]

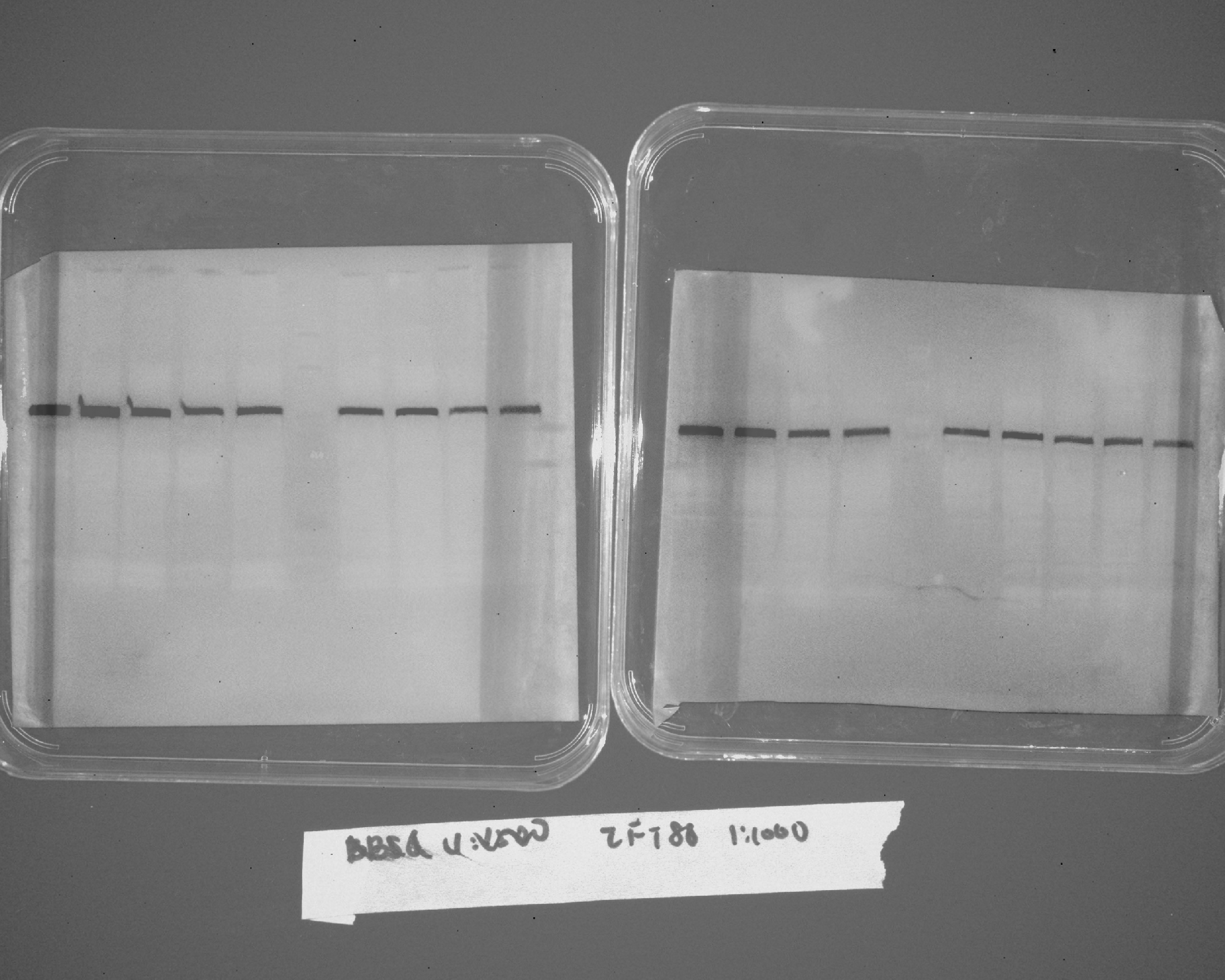

Supplement: Figure 5—source data 5. — RSP stands for reserpine. The size of the protein ladders, IFT88, and relevant sample identity are labeled. [file elife-83205-fig5-data5.zip › Figure 5-source data 5/Figure 5-source data 5_Composite.tif]

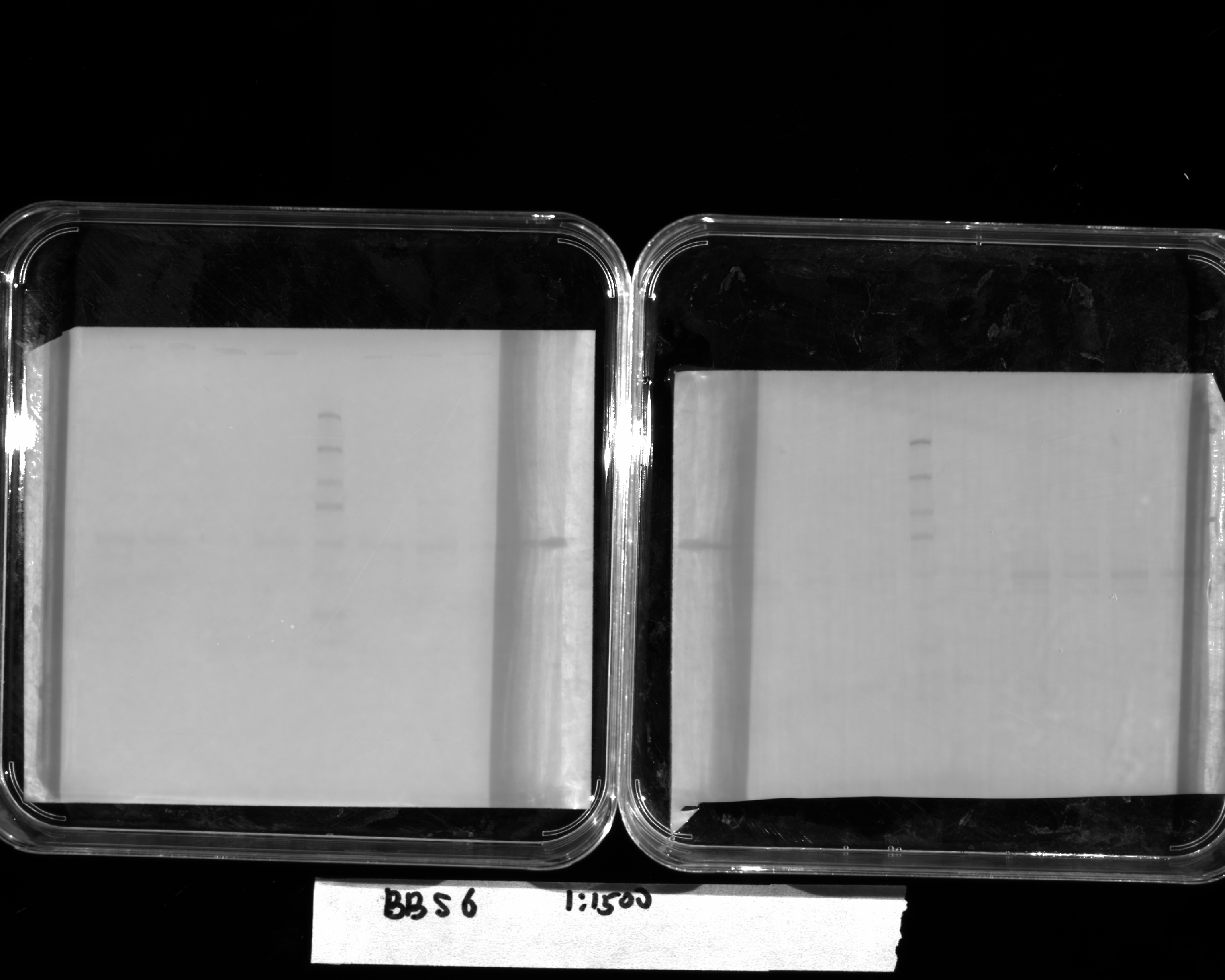

Supplement: Figure 5—source data 6. — RSP stands for reserpine. The size of the protein ladders, BBS6, and relevant sample identity are labeled. [file elife-83205-fig5-data6.zip › Figure 5-source data 6/Figure 5-source data 6_Colorimetric.tif]

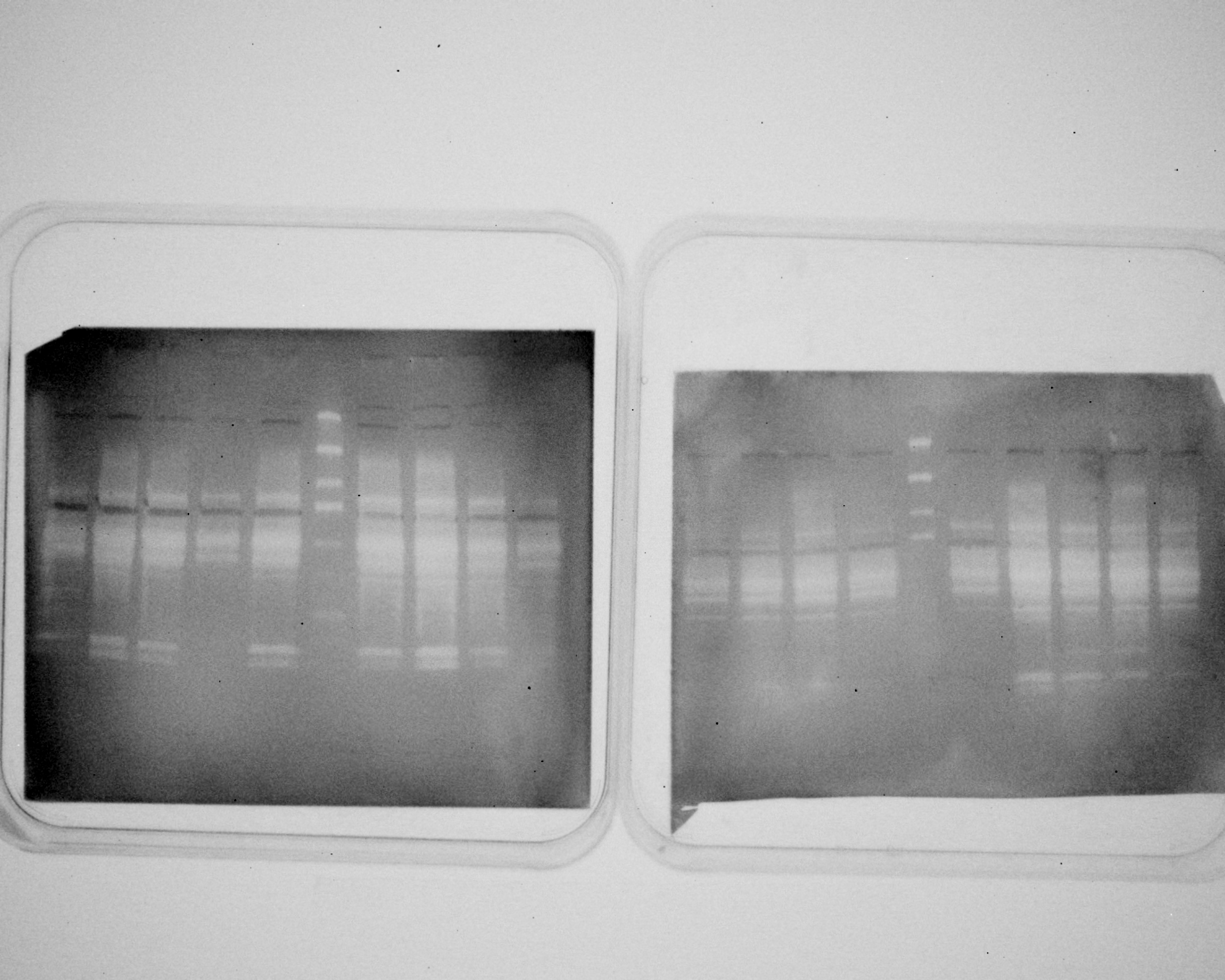

Supplement: Figure 5—source data 6. — RSP stands for reserpine. The size of the protein ladders, BBS6, and relevant sample identity are labeled. [file elife-83205-fig5-data6.zip › Figure 5-source data 6/Figure 5-source data 6_Chemiluminescence.tif]

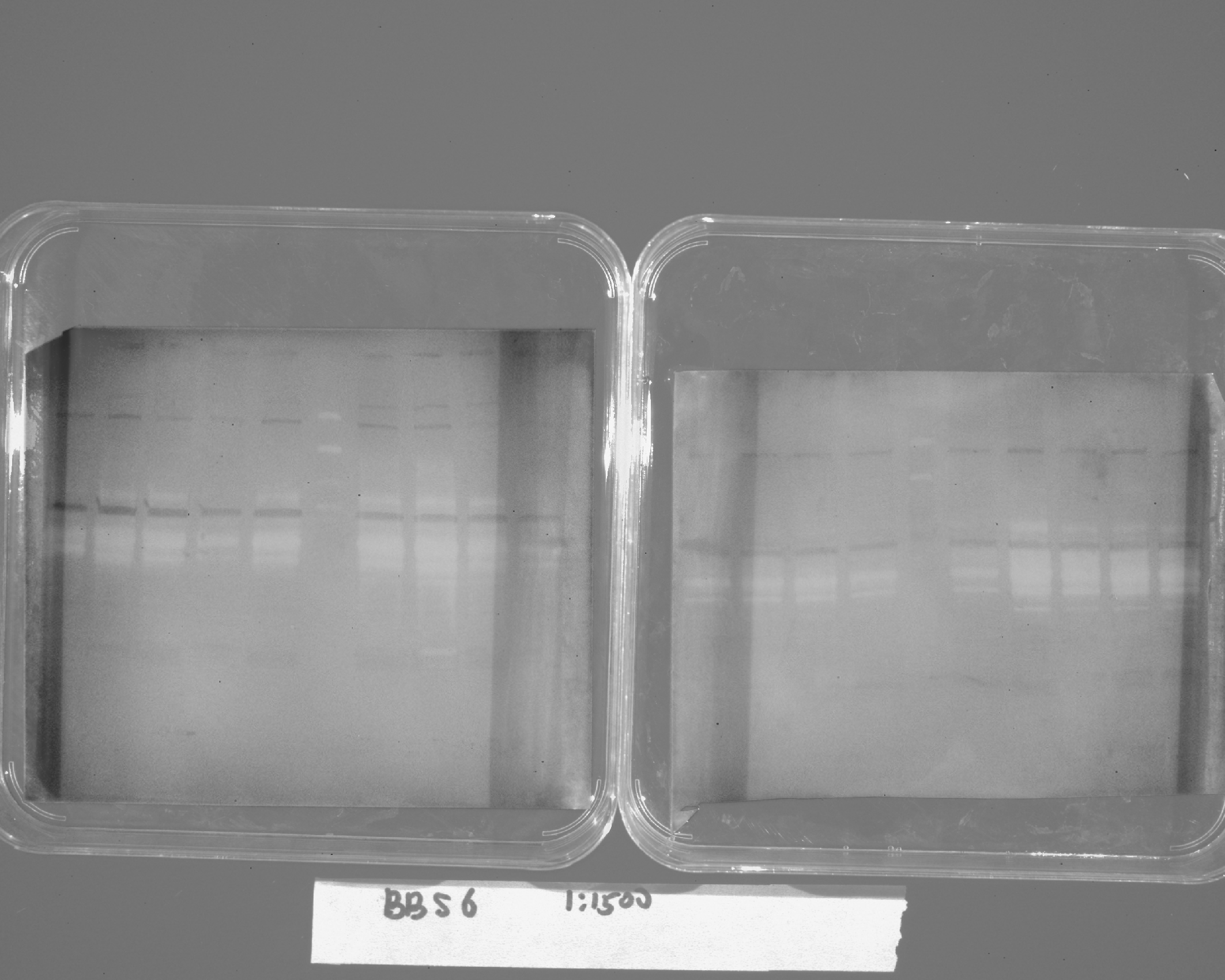

Supplement: Figure 5—source data 6. — RSP stands for reserpine. The size of the protein ladders, BBS6, and relevant sample identity are labeled. [file elife-83205-fig5-data6.zip › Figure 5-source data 6/Figure 5-source data 6_Composite.tif]

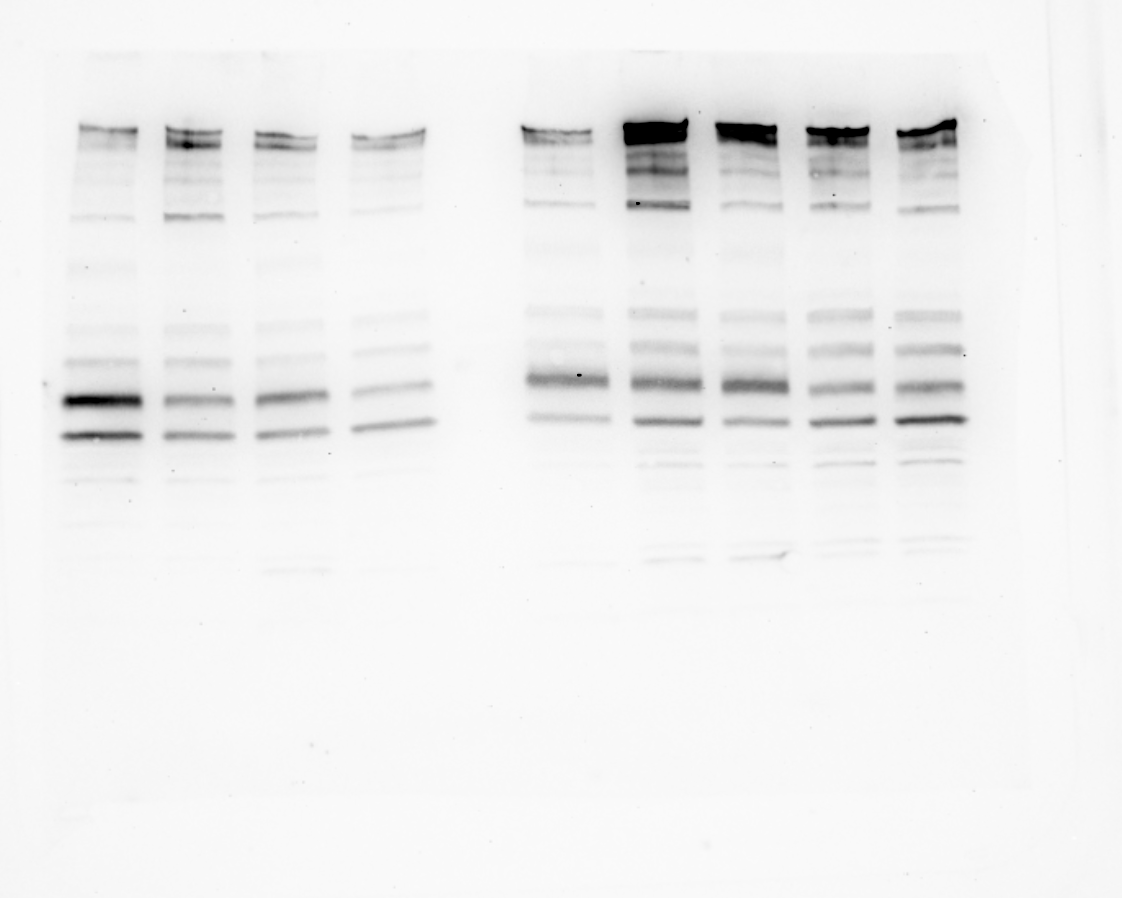

Supplement: Figure 5—source data 7. — RSP stands for reserpine. The size of the protein ladders, CEP164, and relevant sample identity are labeled. [file elife-83205-fig5-data7.zip › Figure 5-source data 7/Figure 5-source data 7_Chemiluminescence.tif]

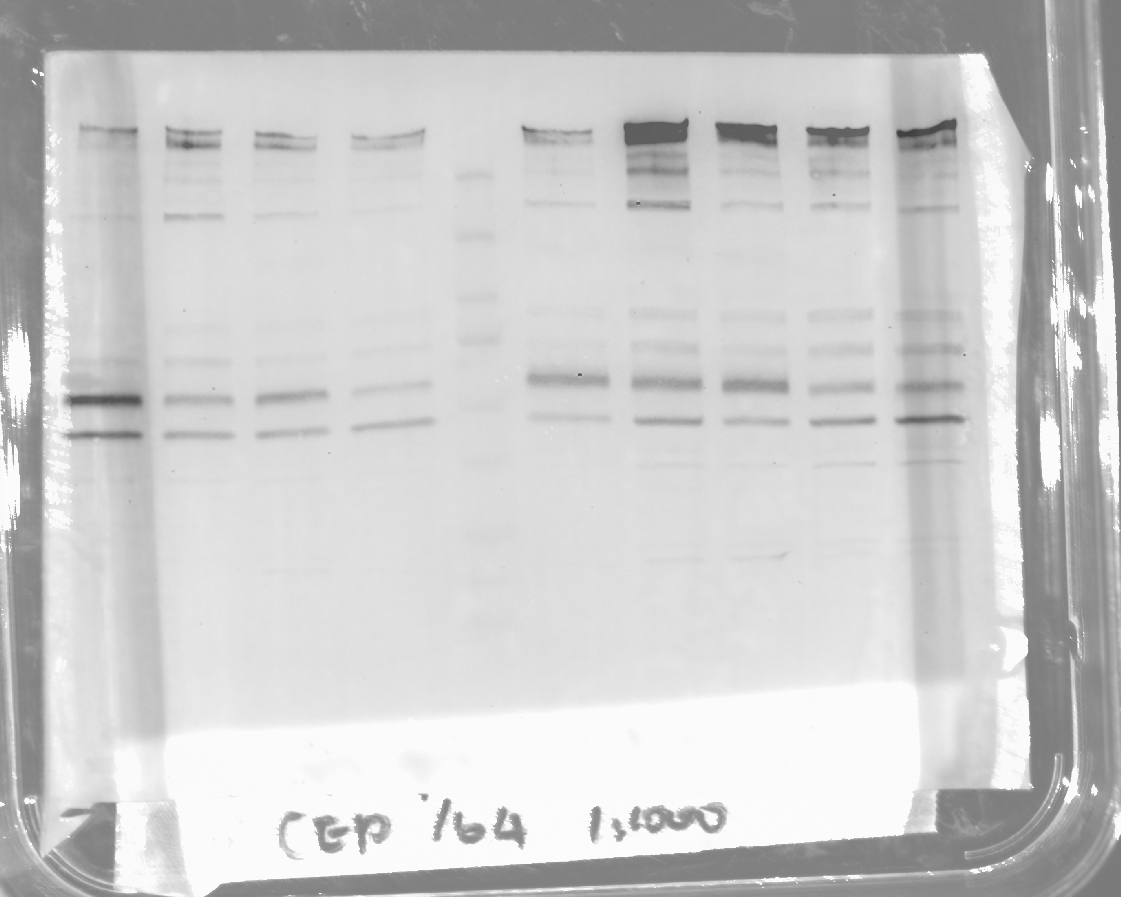

Supplement: Figure 5—source data 7. — RSP stands for reserpine. The size of the protein ladders, CEP164, and relevant sample identity are labeled. [file elife-83205-fig5-data7.zip › Figure 5-source data 7/Figure 5-source data 7_Composite.tif]

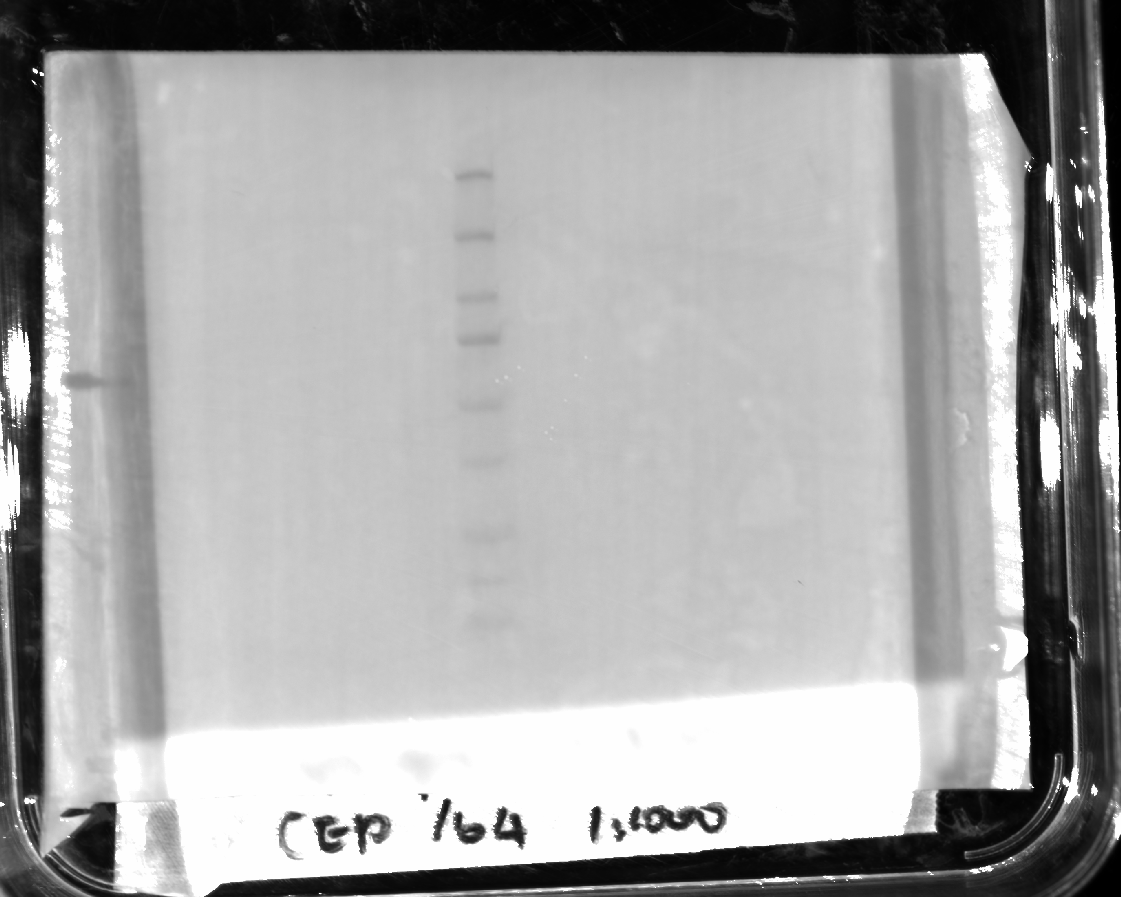

Supplement: Figure 5—source data 7. — RSP stands for reserpine. The size of the protein ladders, CEP164, and relevant sample identity are labeled. [file elife-83205-fig5-data7.zip › Figure 5-source data 7/Figure 5-source data 7_Colorimetric.tif]

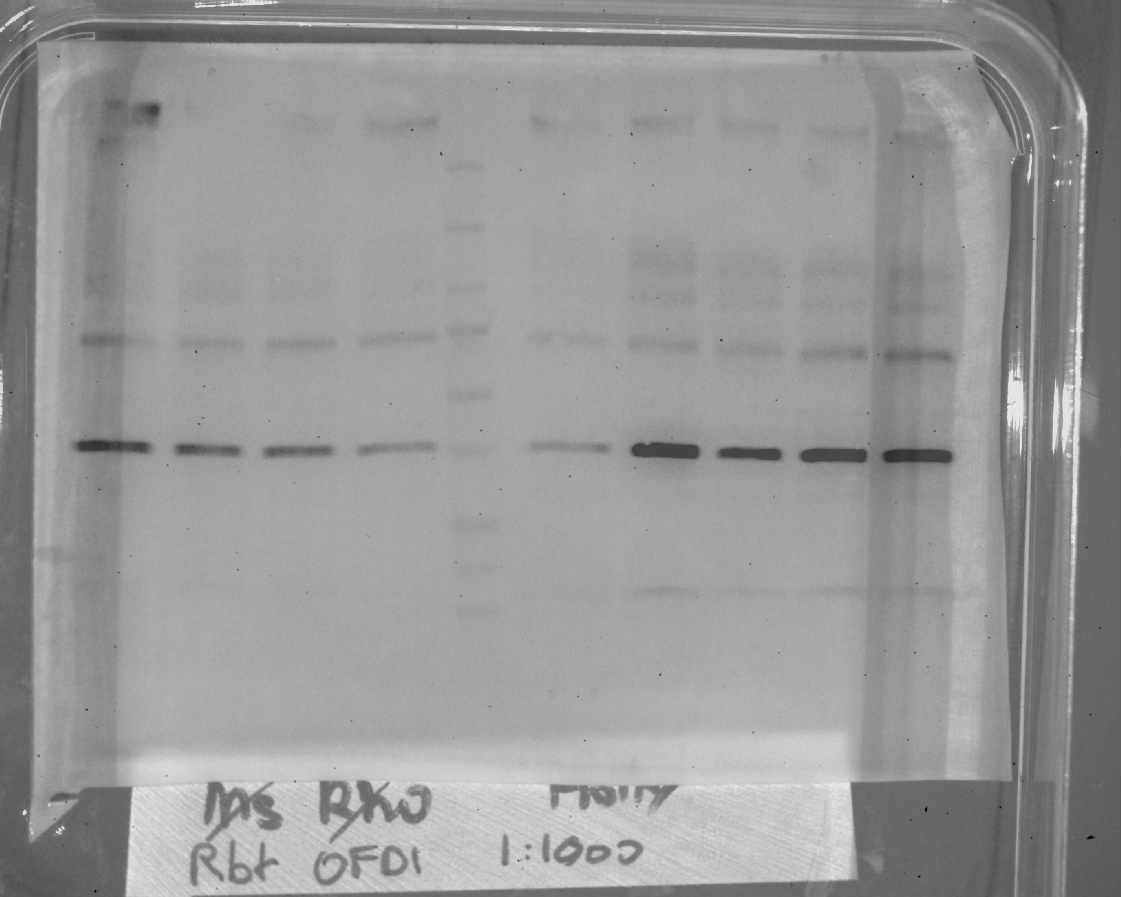

Supplement: Figure 5—source data 8. — RSP stands for reserpine. The size of the protein ladders, OFD1, and relevant sample identity are labeled. [file elife-83205-fig5-data8.zip › Figure 5-source data 8/Figure 5-source data 8_Composite.tif]

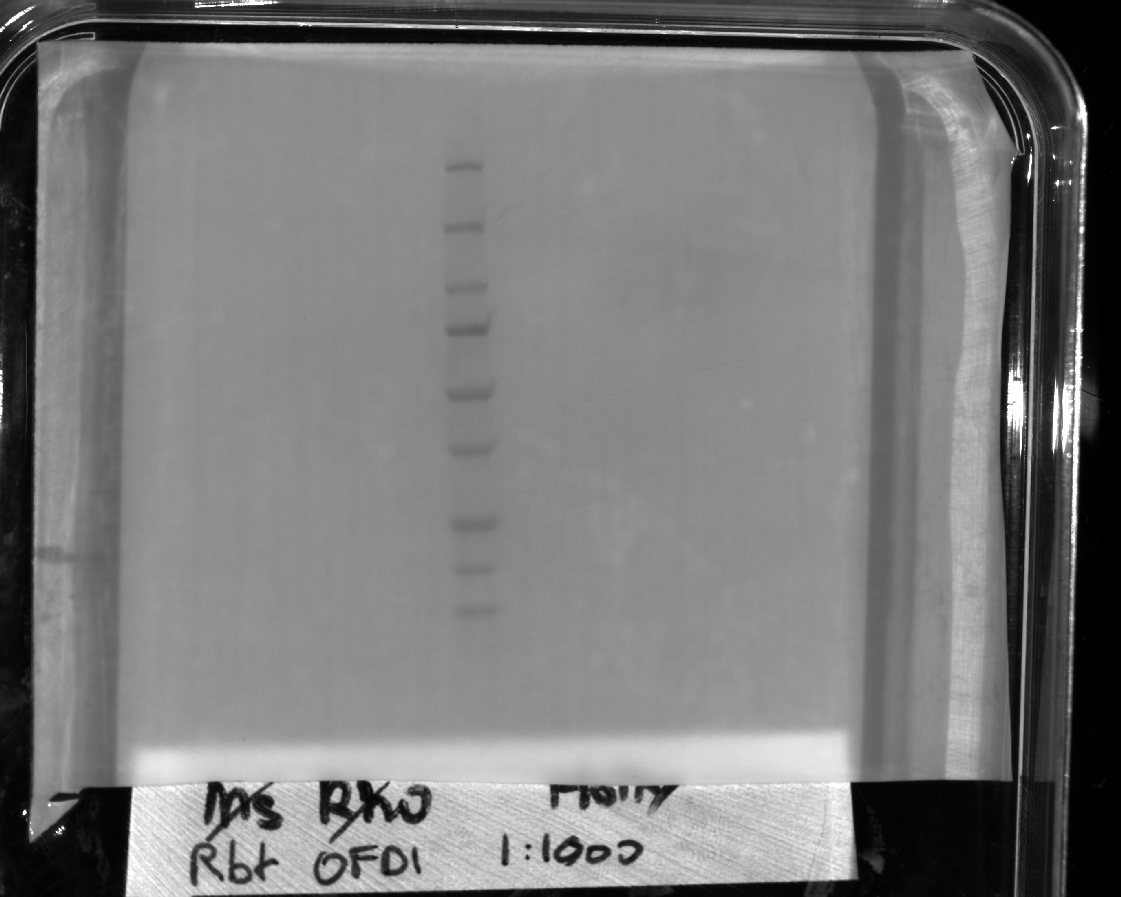

Supplement: Figure 5—source data 8. — RSP stands for reserpine. The size of the protein ladders, OFD1, and relevant sample identity are labeled. [file elife-83205-fig5-data8.zip › Figure 5-source data 8/Figure 5-source data 8_Colorimetric.tif]

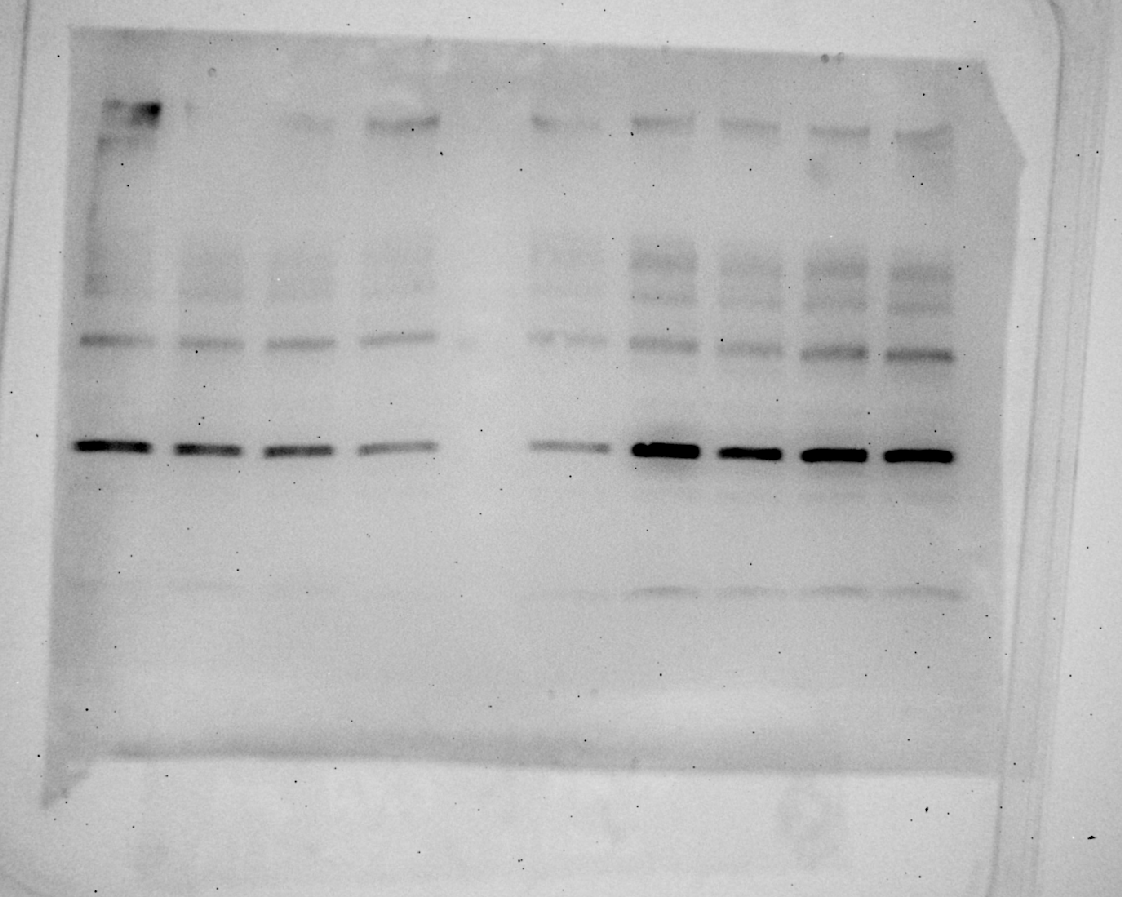

Supplement: Figure 5—source data 8. — RSP stands for reserpine. The size of the protein ladders, OFD1, and relevant sample identity are labeled. [file elife-83205-fig5-data8.zip › Figure 5-source data 8/Figure 5-source data 8_Chemiluminescence.tif]

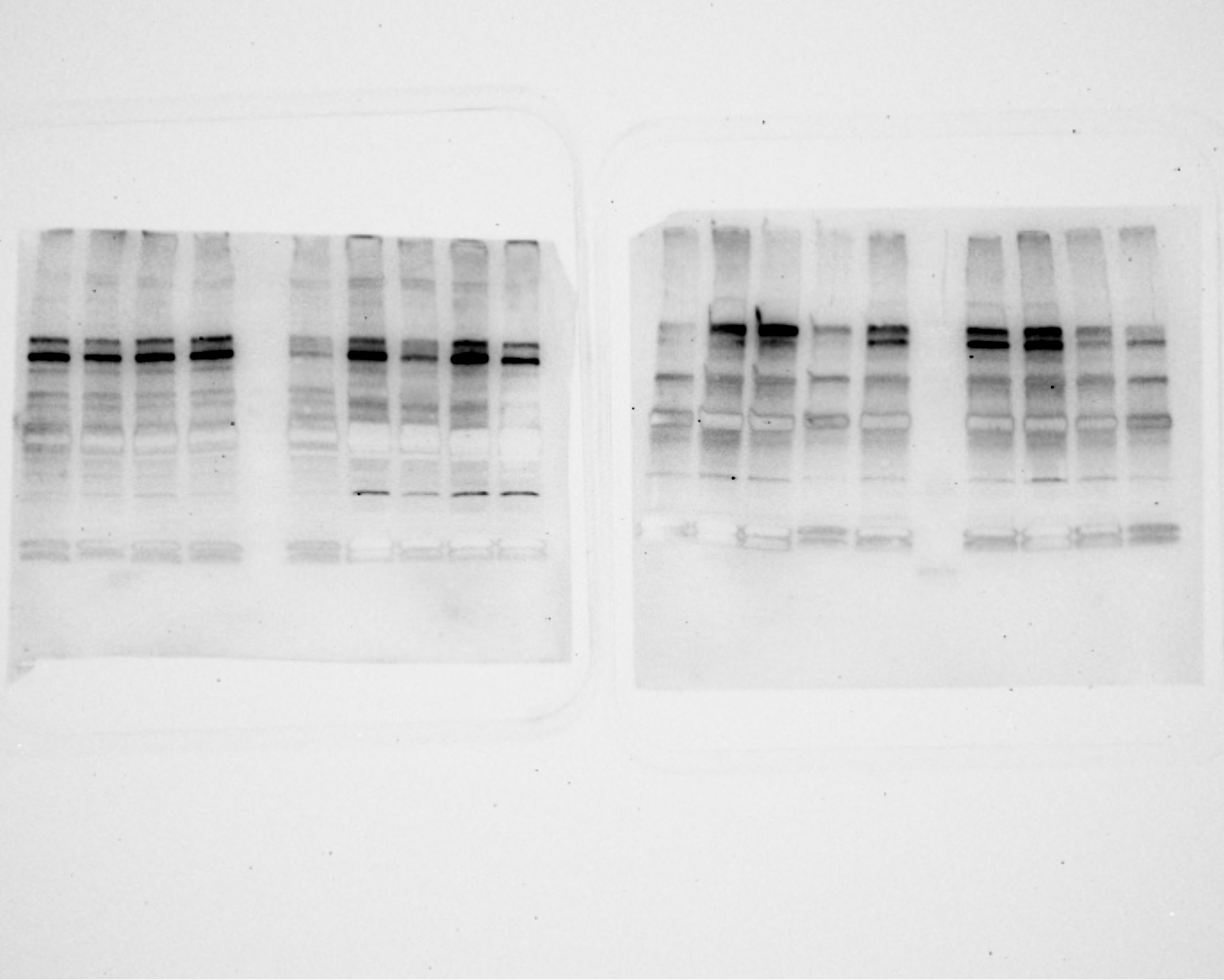

Supplement: Figure 5—source data 9. — RSP stands for reserpine. The size of the protein ladders, HDAC6, and relevant sample identity are labeled. [file elife-83205-fig5-data9.zip › Figure 5-source data 9/Figure 5-source data 9_Chemiluminescence.tif]

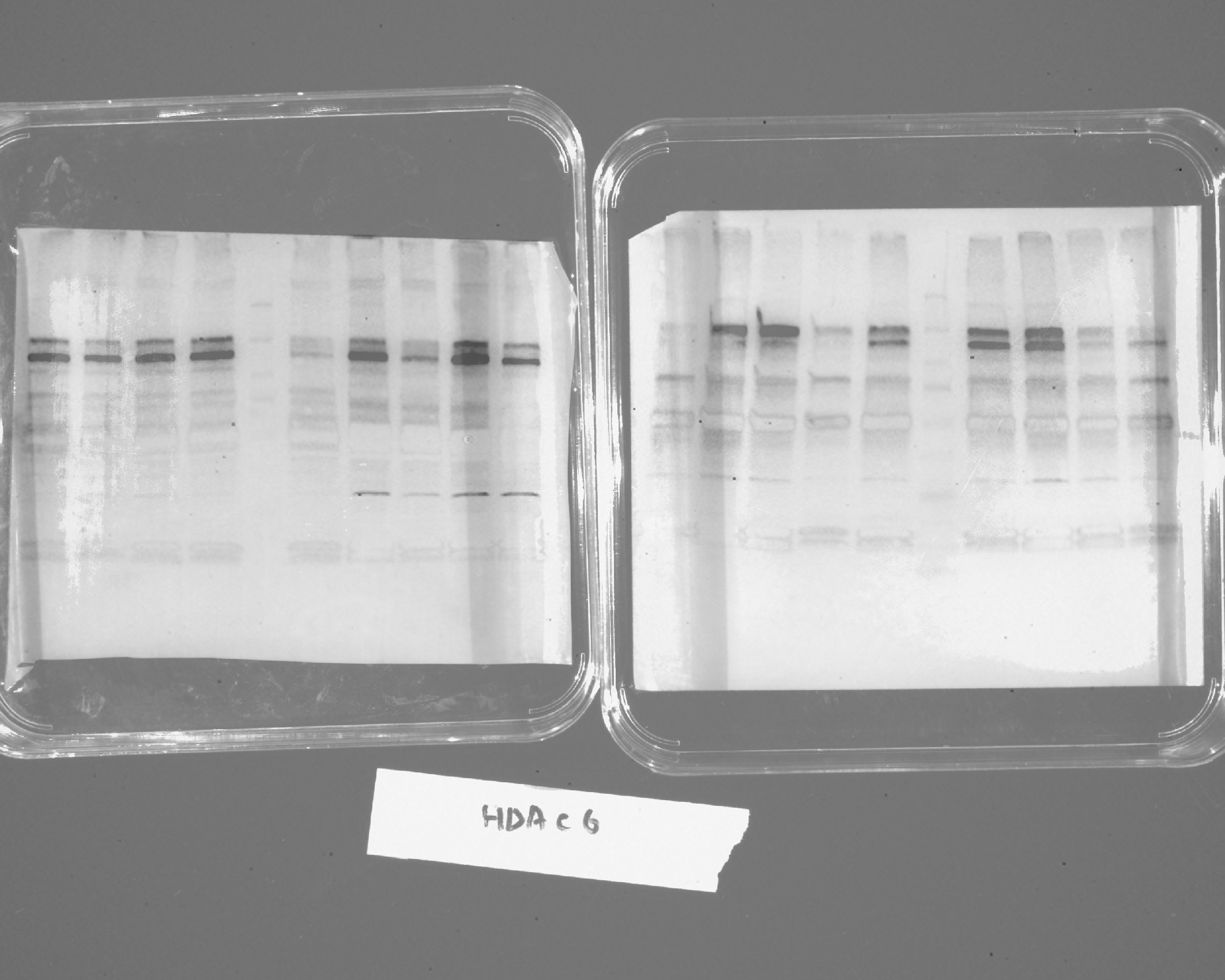

Supplement: Figure 5—source data 9. — RSP stands for reserpine. The size of the protein ladders, HDAC6, and relevant sample identity are labeled. [file elife-83205-fig5-data9.zip › Figure 5-source data 9/Figure 5-source data 9_Composite.tif]

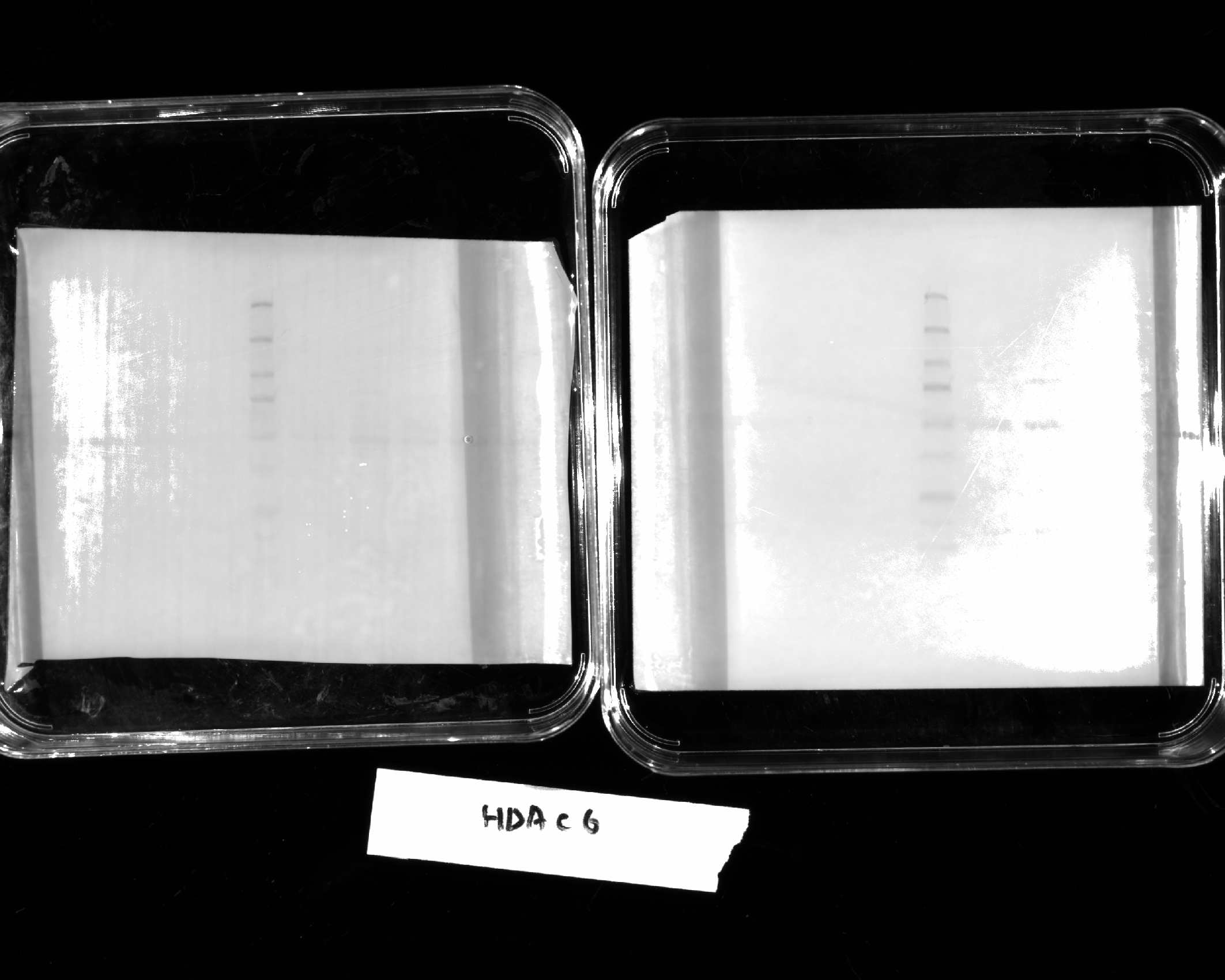

Supplement: Figure 5—source data 9. — RSP stands for reserpine. The size of the protein ladders, HDAC6, and relevant sample identity are labeled. [file elife-83205-fig5-data9.zip › Figure 5-source data 9/Figure 5-source data 9_Colorimetric.tif]

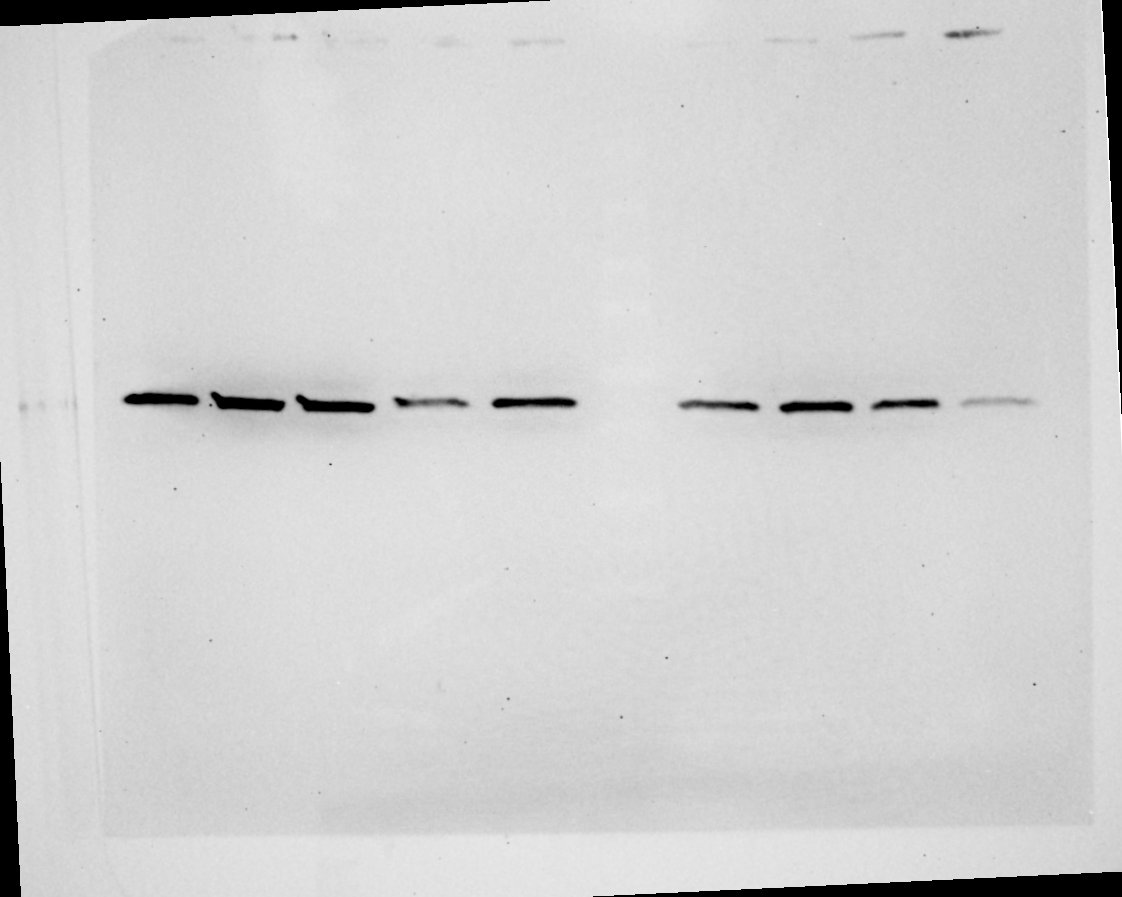

Supplement: Figure 5—source data 10. — RSP stands for reserpine. The size of the protein ladders, β-Actin, and relevant sample identity are labeled. [file elife-83205-fig5-data10.zip › Figure 5-source data 10/Figure 5-source data 10_Chemiluminescence.tif]

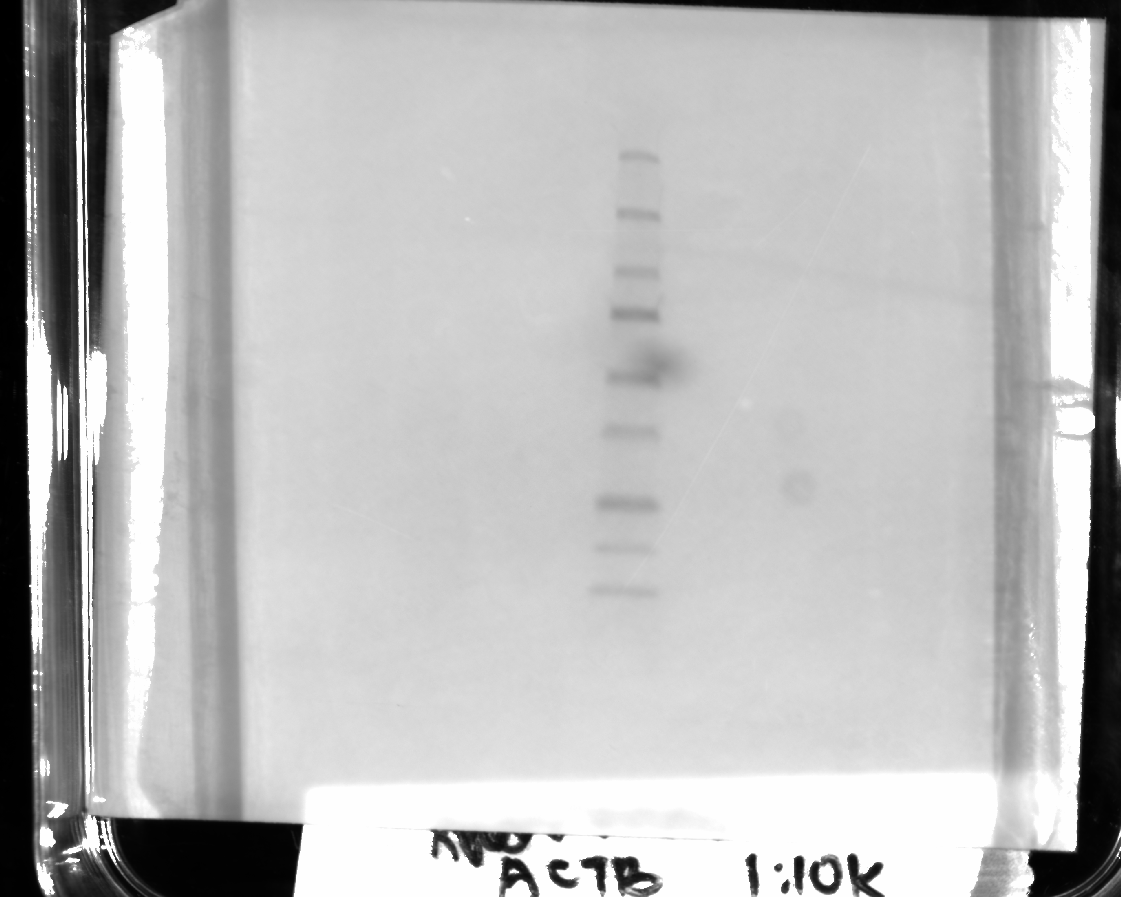

Supplement: Figure 5—source data 10. — RSP stands for reserpine. The size of the protein ladders, β-Actin, and relevant sample identity are labeled. [file elife-83205-fig5-data10.zip › Figure 5-source data 10/Figure 5-source data 10_Colorimetric.tif]

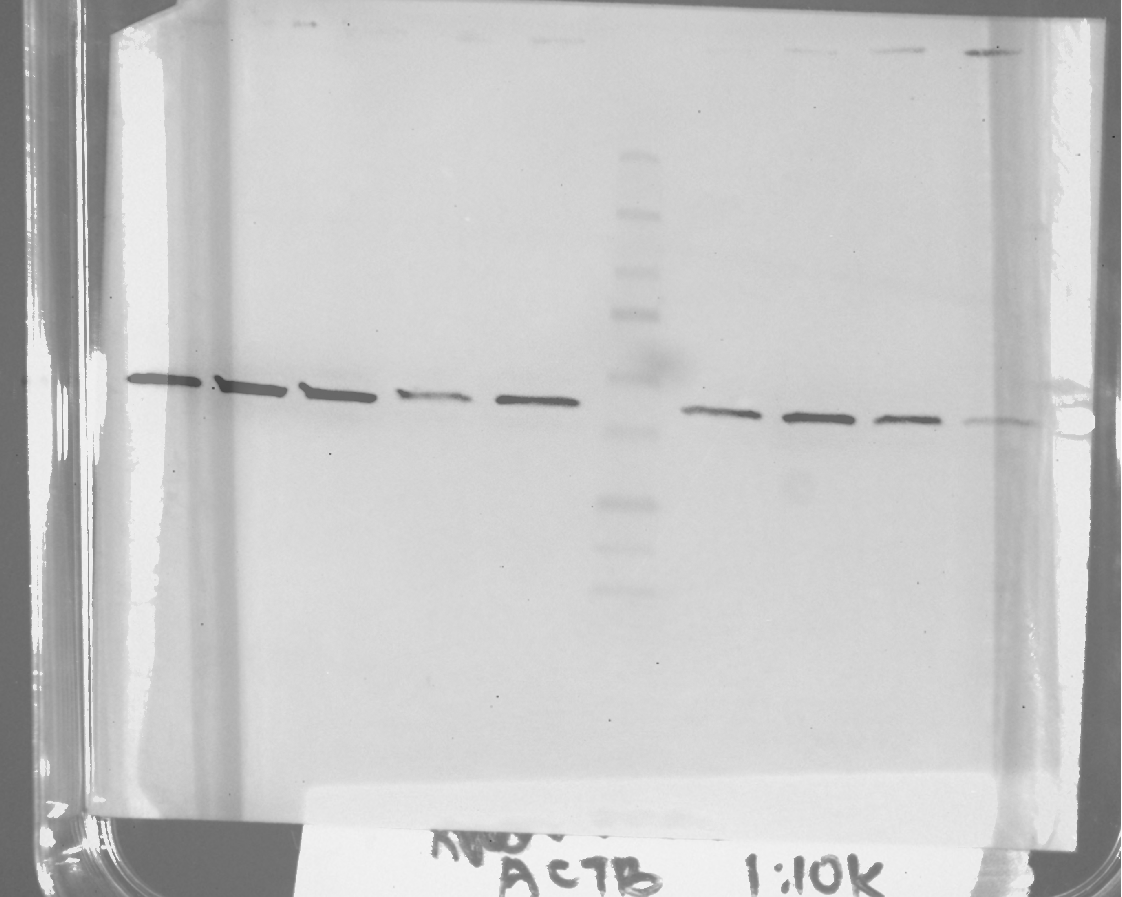

Supplement: Figure 5—source data 10. — RSP stands for reserpine. The size of the protein ladders, β-Actin, and relevant sample identity are labeled. [file elife-83205-fig5-data10.zip › Figure 5-source data 10/Figure 5-source data 10_Composite.tif]

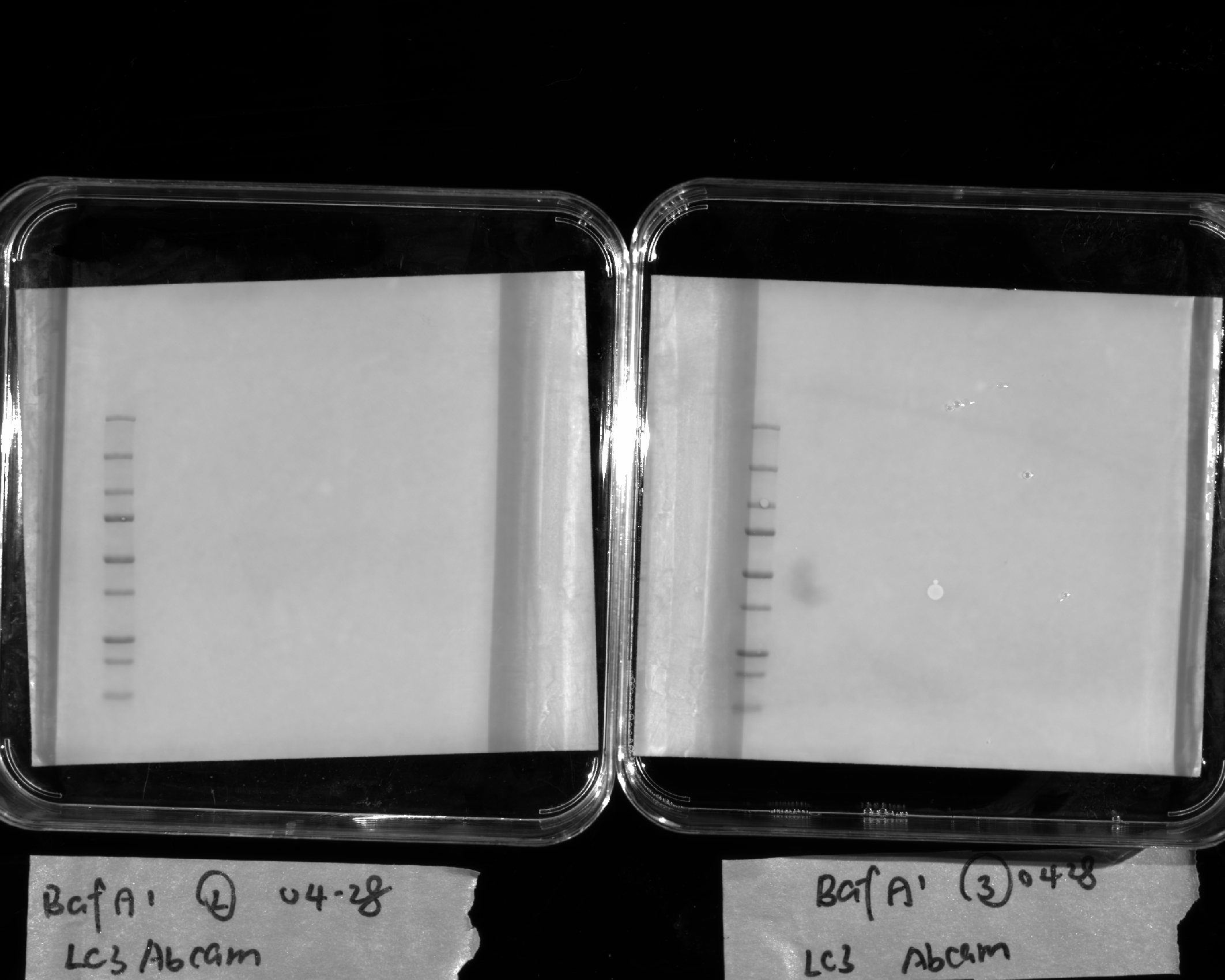

Supplement: Figure 5—figure supplement 2—source data 1. — BafA1 stands for Bafilomycin A1. The size of the protein ladders, LC3, and relevant sample identity are labeled. [file elife-83205-fig5-figsupp2-data1.zip › Figure 5-figure supplement 2-source data 1/Figure 5-figure supplement 2-source data 1_Colorimetric.tif]

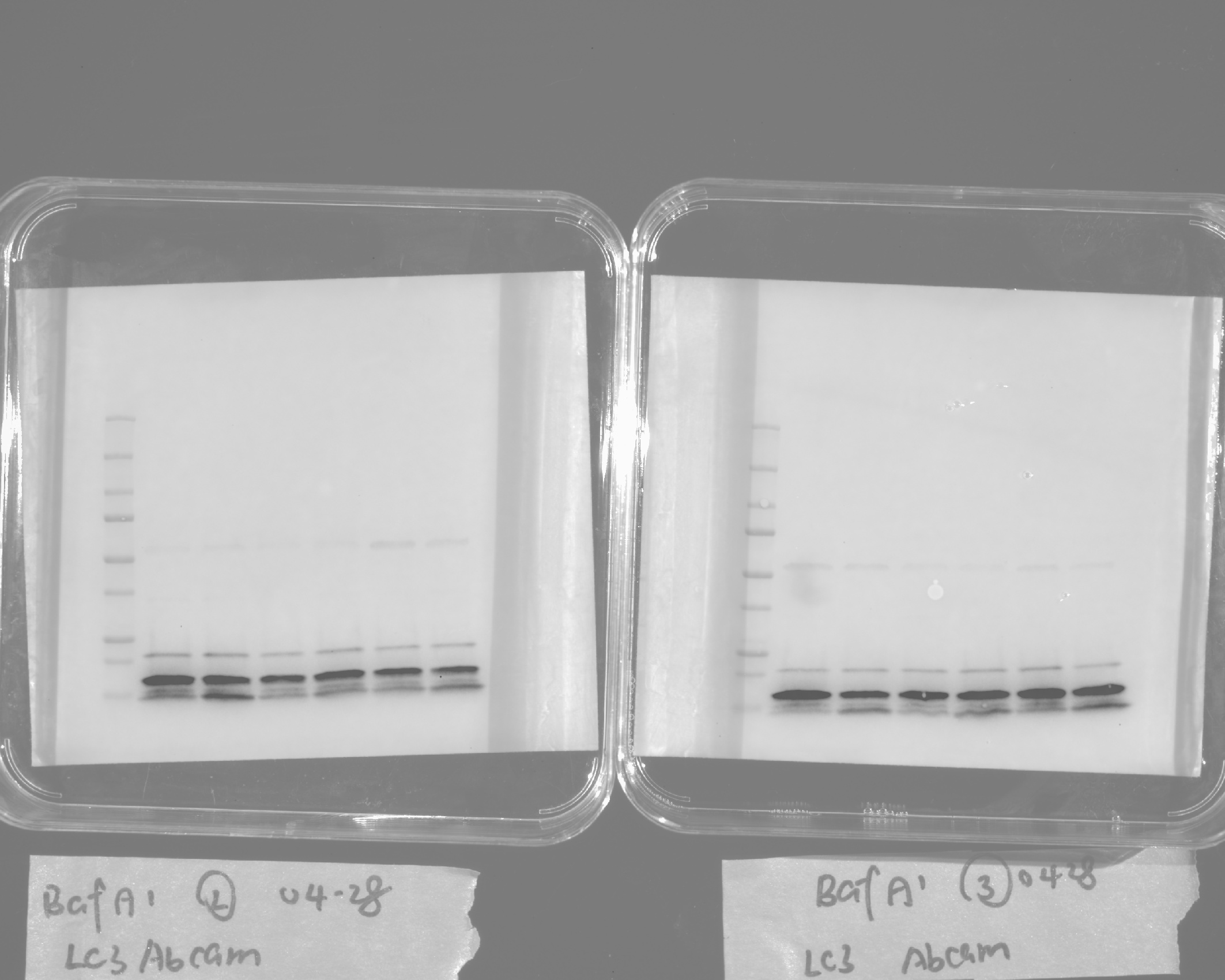

Supplement: Figure 5—figure supplement 2—source data 1. — BafA1 stands for Bafilomycin A1. The size of the protein ladders, LC3, and relevant sample identity are labeled. [file elife-83205-fig5-figsupp2-data1.zip › Figure 5-figure supplement 2-source data 1/Figure 5-figure supplement 2-source data 1_Composite.tif]

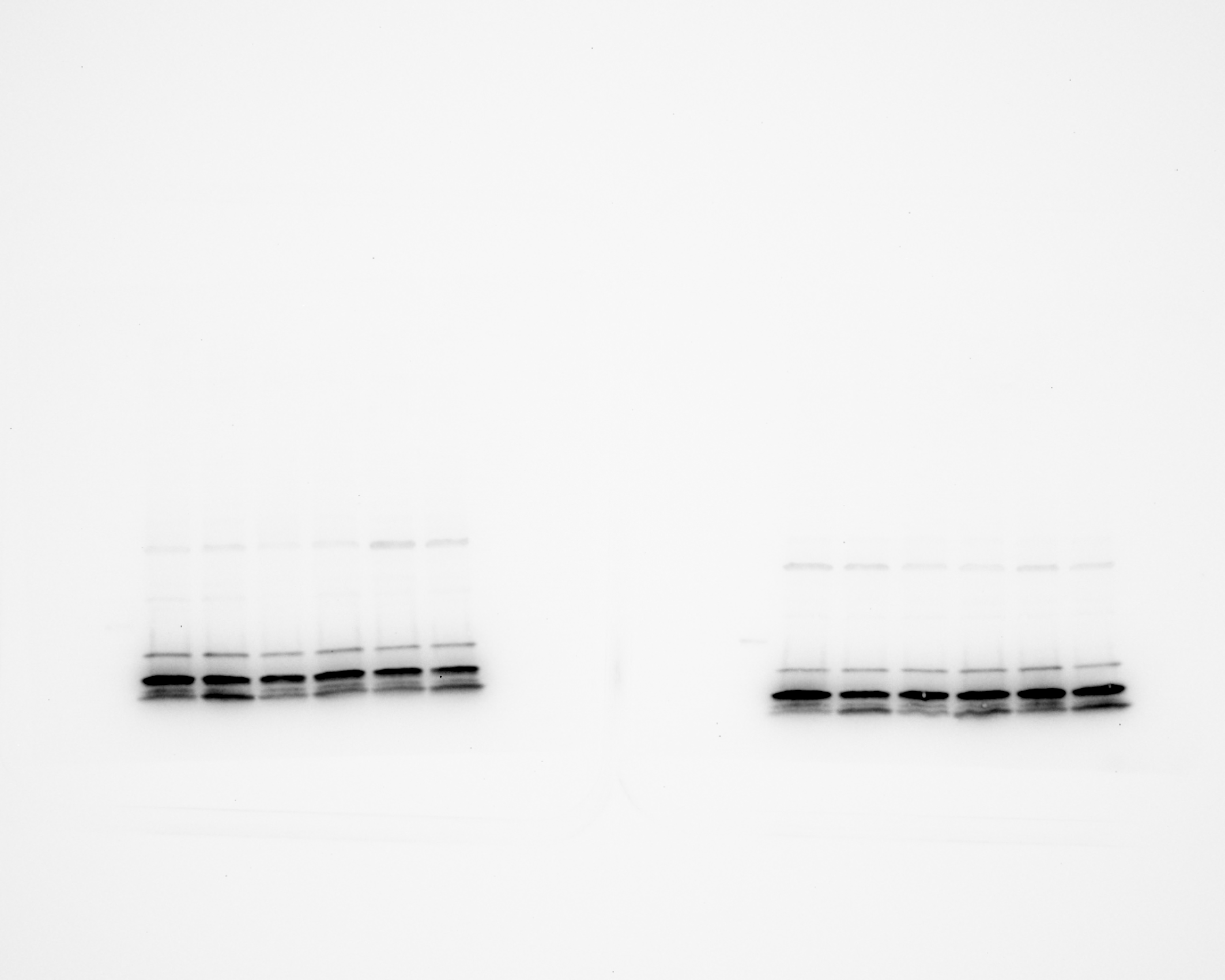

Supplement: Figure 5—figure supplement 2—source data 1. — BafA1 stands for Bafilomycin A1. The size of the protein ladders, LC3, and relevant sample identity are labeled. [file elife-83205-fig5-figsupp2-data1.zip › Figure 5-figure supplement 2-source data 1/Figure 5-figure supplement 2-source data 1_Chemiluminescence.tif]

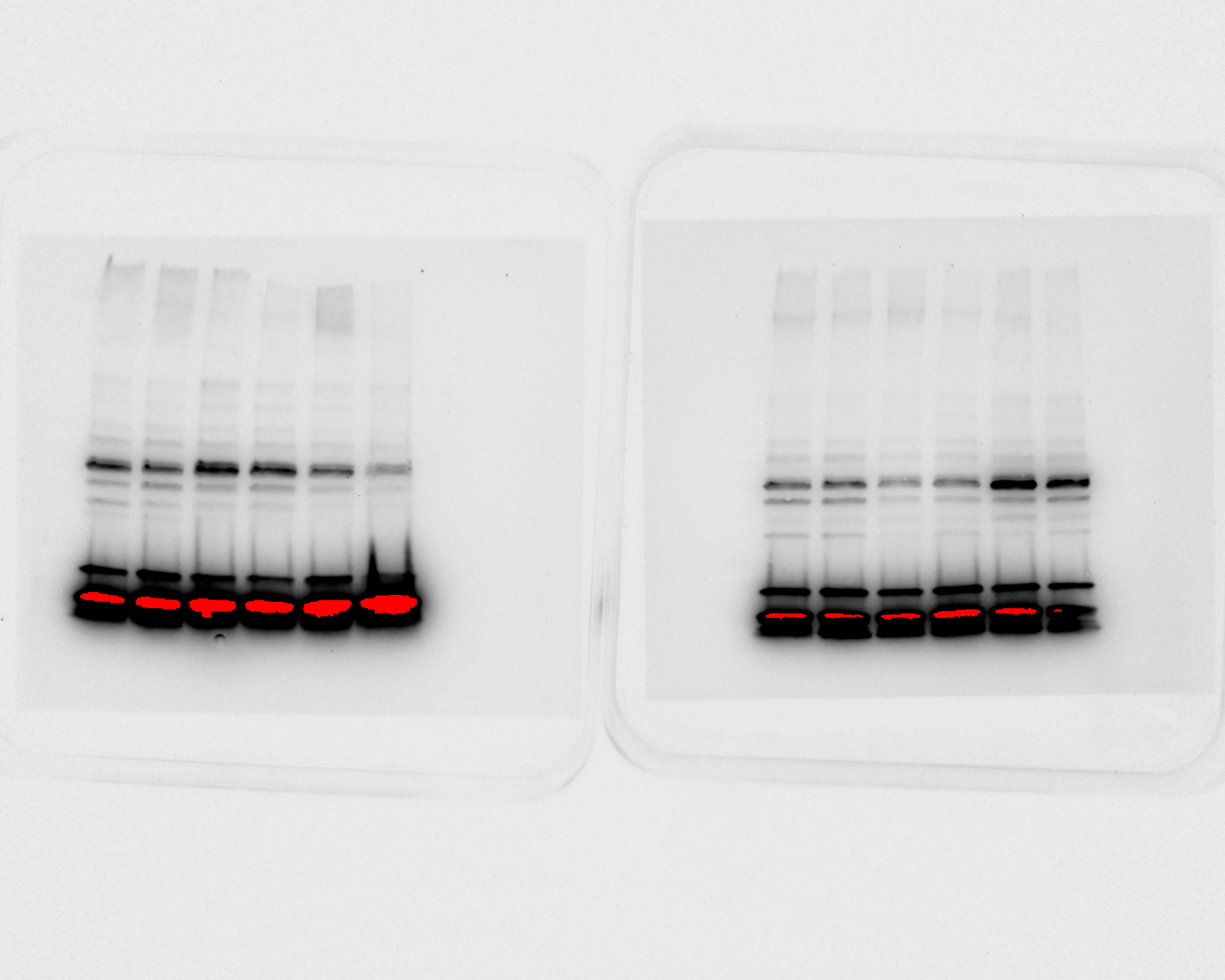

Supplement: Figure 5—figure supplement 2—source data 2. — BafA1 stands for Bafilomycin A1. The size of the protein ladders, p62, and relevant sample identity are labeled. [file elife-83205-fig5-figsupp2-data2.zip › Figure 5-figure supplement 2-source data 2/Figure 5-figure supplement 2-source data 2_Chemiluminescence.tif]

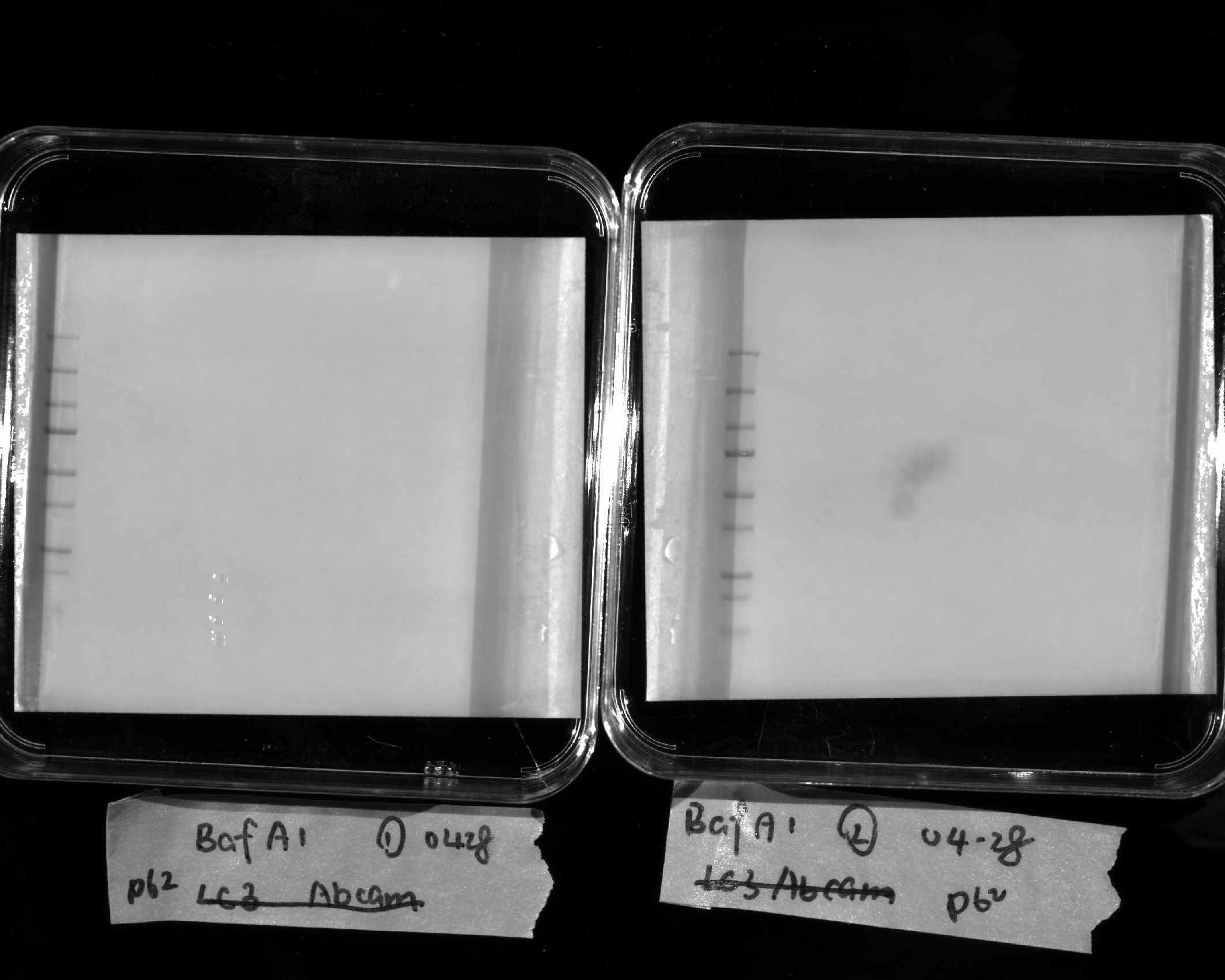

Supplement: Figure 5—figure supplement 2—source data 2. — BafA1 stands for Bafilomycin A1. The size of the protein ladders, p62, and relevant sample identity are labeled. [file elife-83205-fig5-figsupp2-data2.zip › Figure 5-figure supplement 2-source data 2/Figure 5-figure supplement 2_ source data 2_Colorimetric.tif]

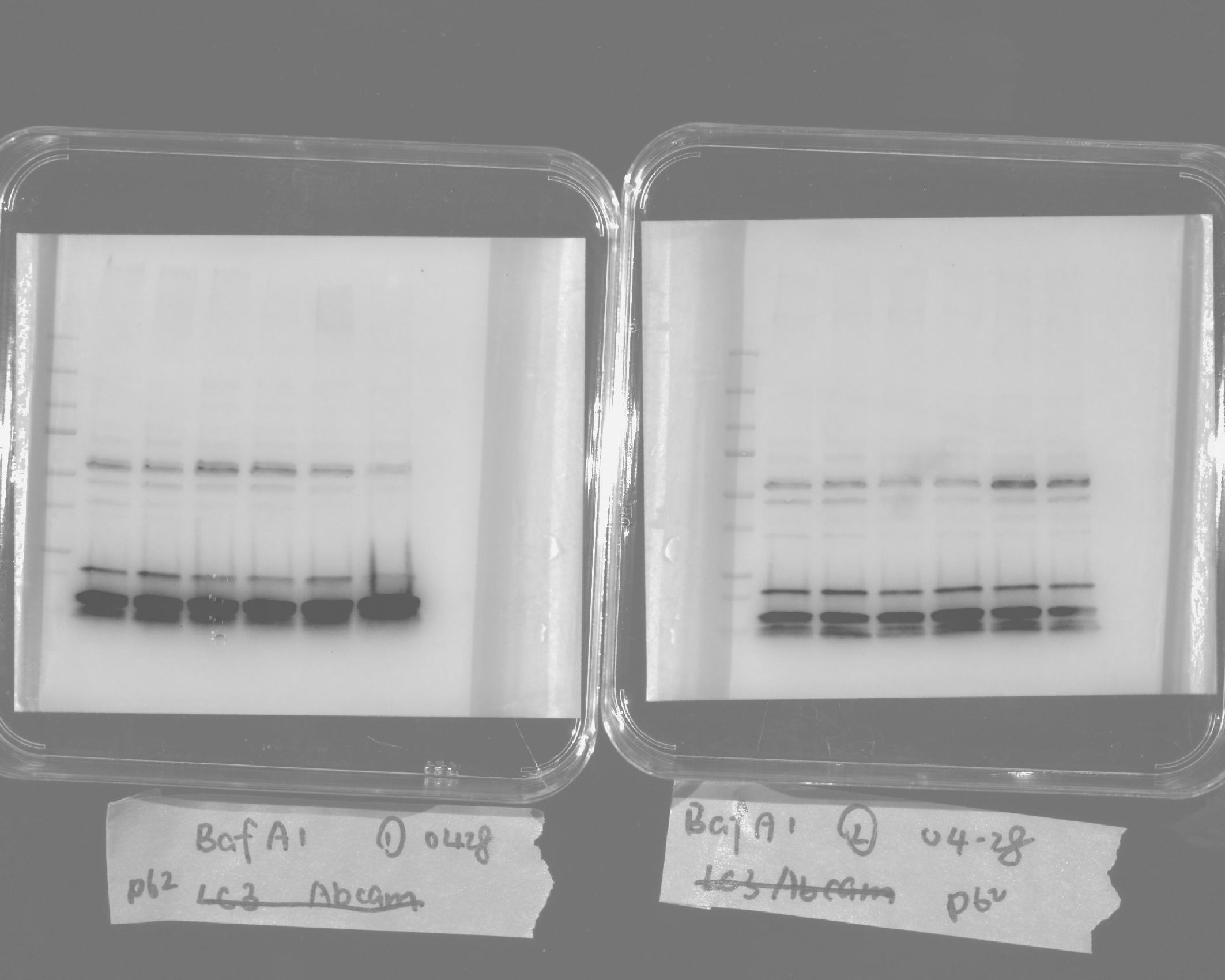

Supplement: Figure 5—figure supplement 2—source data 2. — BafA1 stands for Bafilomycin A1. The size of the protein ladders, p62, and relevant sample identity are labeled. [file elife-83205-fig5-figsupp2-data2.zip › Figure 5-figure supplement 2-source data 2/Figure 5-figure supplement 2-source data 2_Composite.tif]

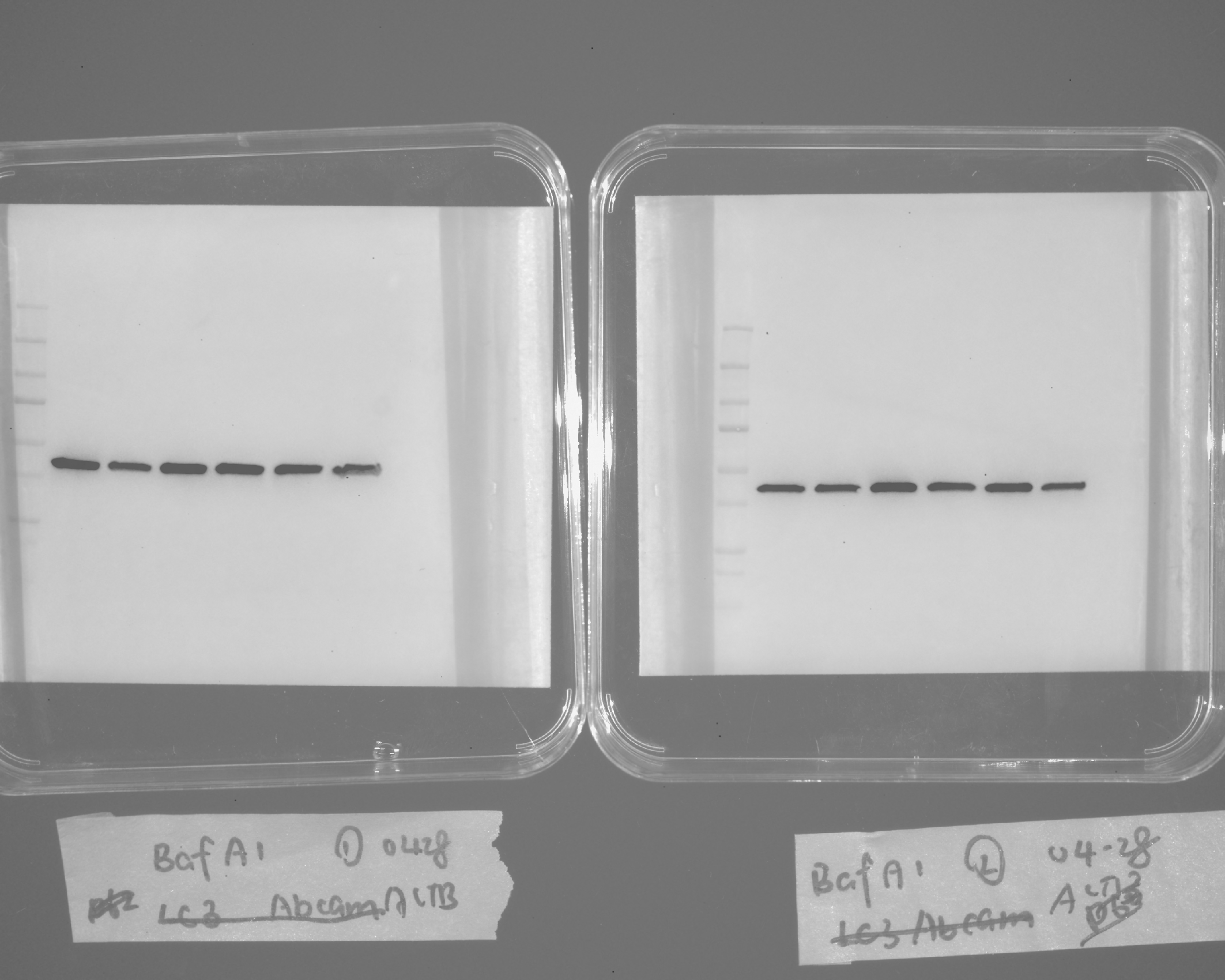

Supplement: Figure 5—figure supplement 2—source data 3. — BafA1 stands for Bafilomycin A1. The size of the protein ladders, b-Actin, and relevant sample identity are labeled. [file elife-83205-fig5-figsupp2-data3.zip › Figure 5-figure supplement 2-source data 3/Figure 5-figure supplement 2-source data 3_Composite.tif]

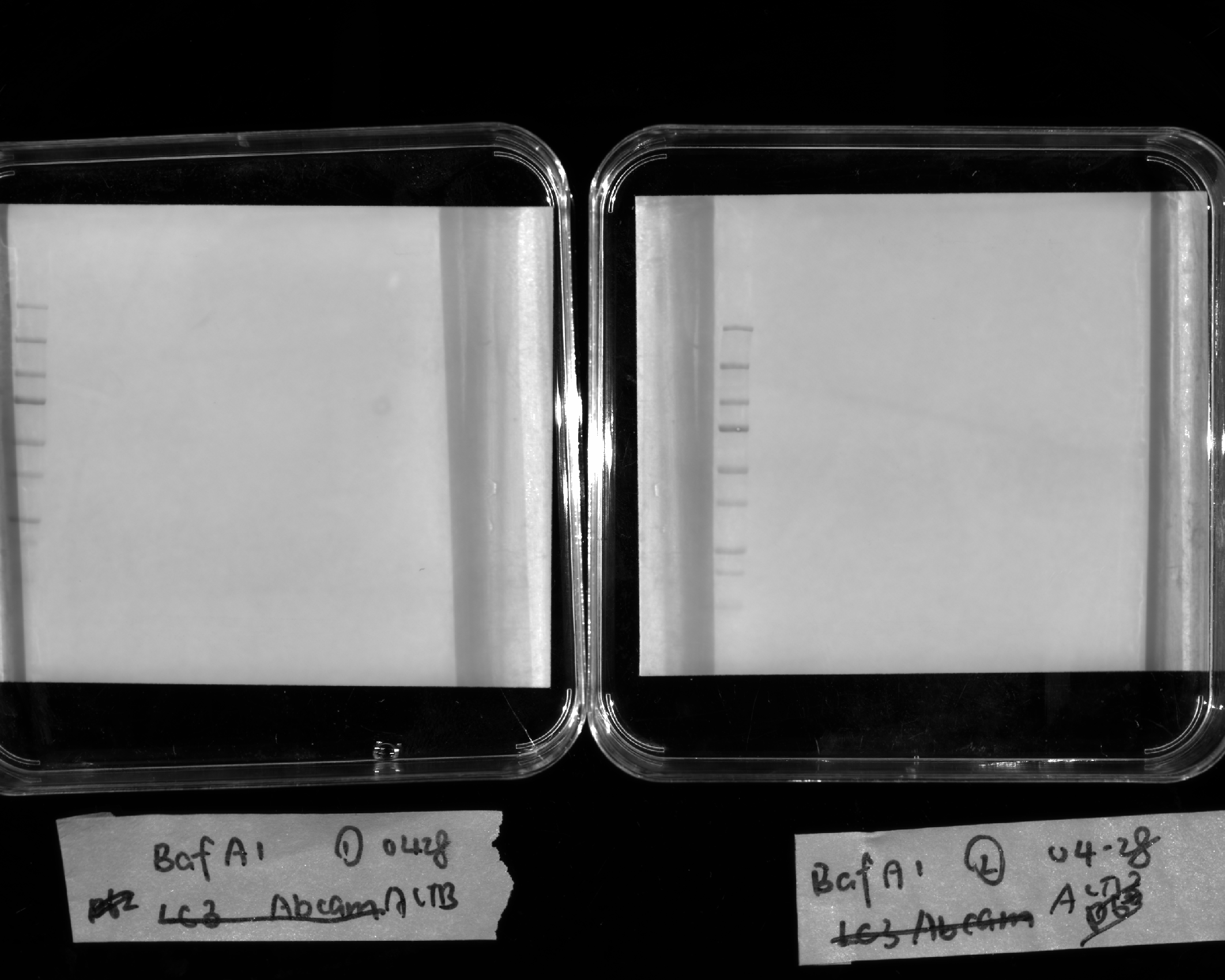

Supplement: Figure 5—figure supplement 2—source data 3. — BafA1 stands for Bafilomycin A1. The size of the protein ladders, b-Actin, and relevant sample identity are labeled. [file elife-83205-fig5-figsupp2-data3.zip › Figure 5-figure supplement 2-source data 3/Figure 5-figure supplement 2-source data 3_Colorimetric.tif]

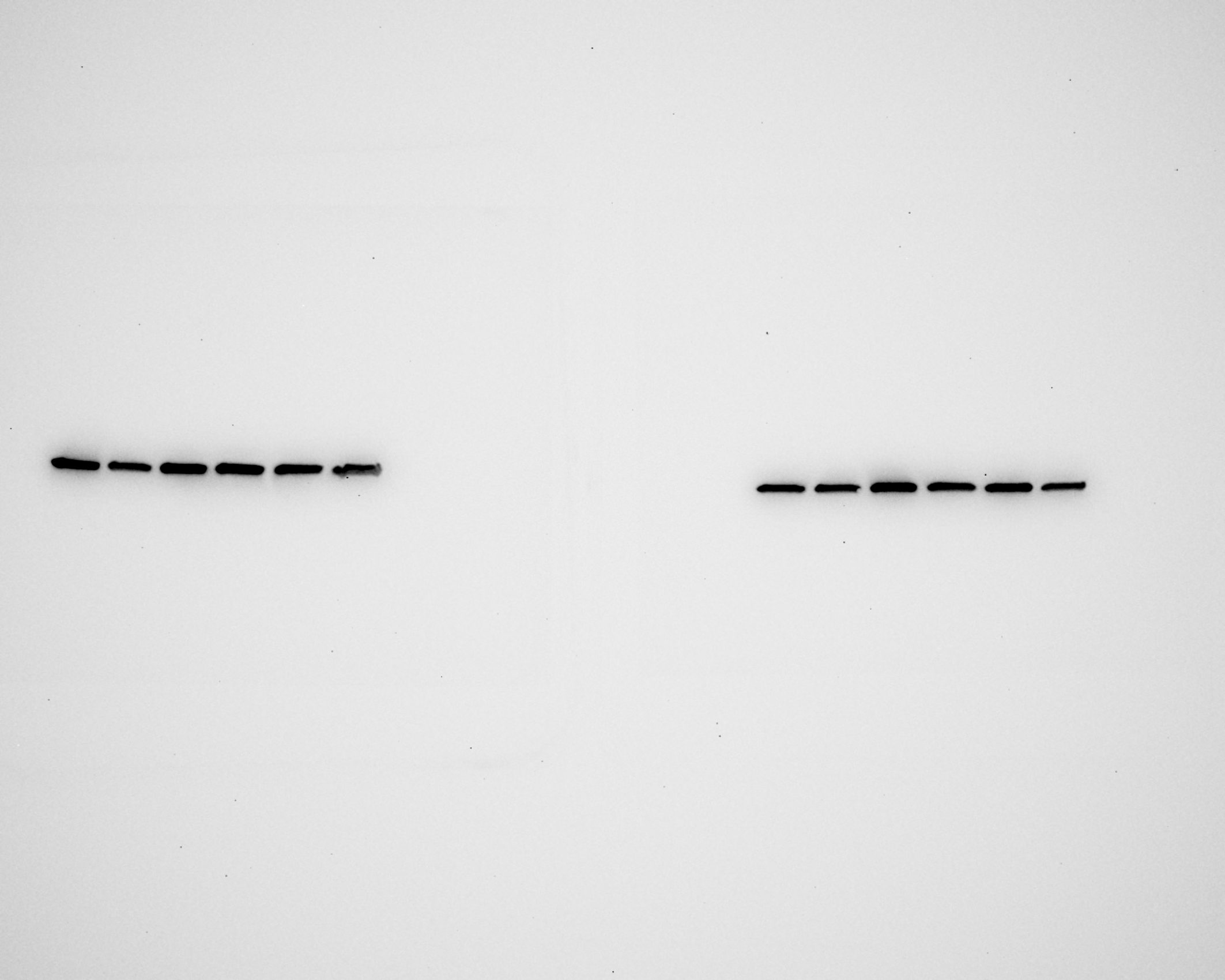

Supplement: Figure 5—figure supplement 2—source data 3. — BafA1 stands for Bafilomycin A1. The size of the protein ladders, b-Actin, and relevant sample identity are labeled. [file elife-83205-fig5-figsupp2-data3.zip › Figure 5-figure supplement 2-source data 3/Figure 5-figure supplement 2-source data 3_Chemiluminescence.tif]

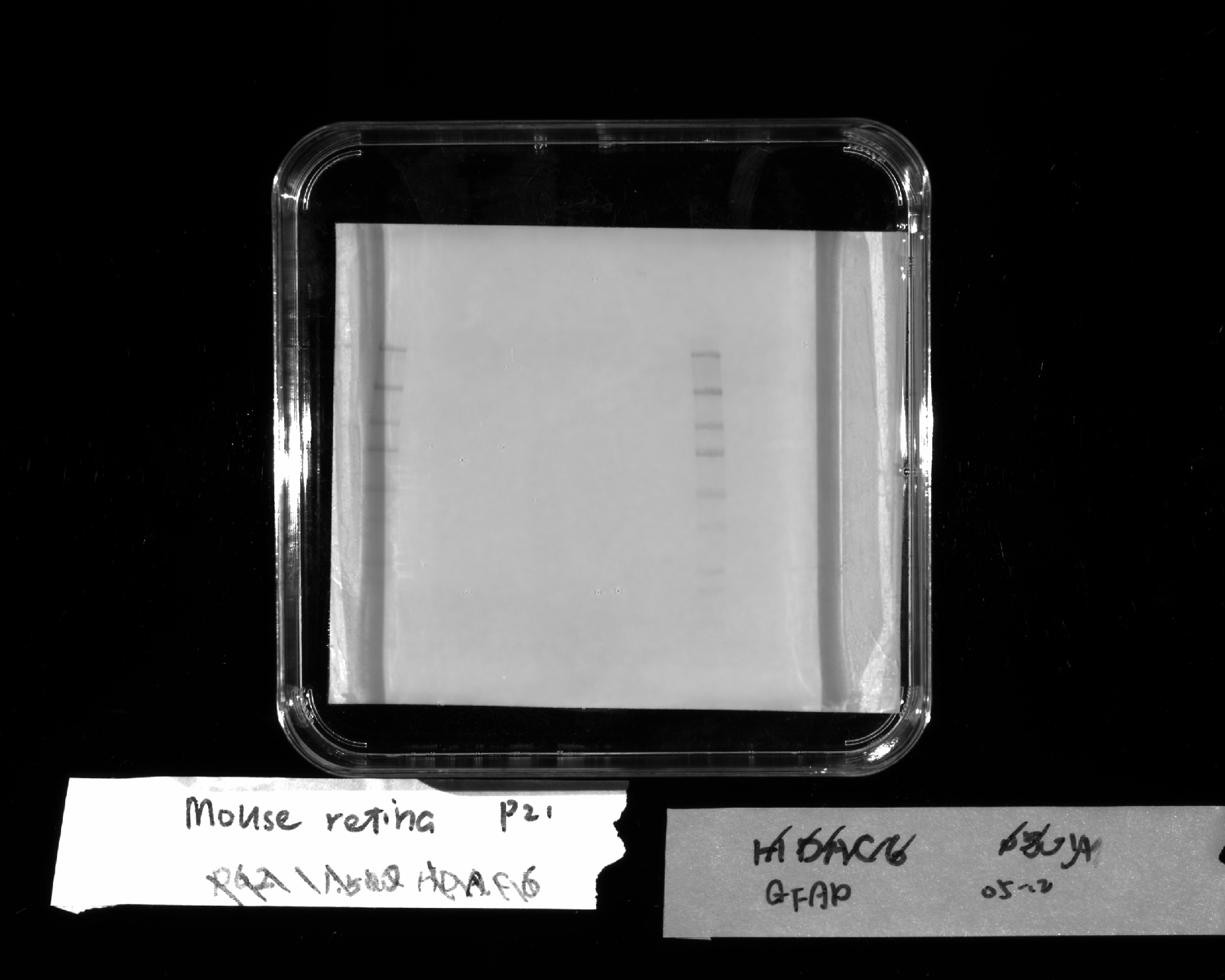

Supplement: Figure 6—figure supplement 3—source data 1. — The size of the protein ladders, Gfap, and relevant sample identity are labeled. [file elife-83205-fig6-figsupp3-data1.zip › Figure 6-figure supplement 3-source data 1/Figure 6-figure supplement 3-source data 1_Colorimetric.tif]

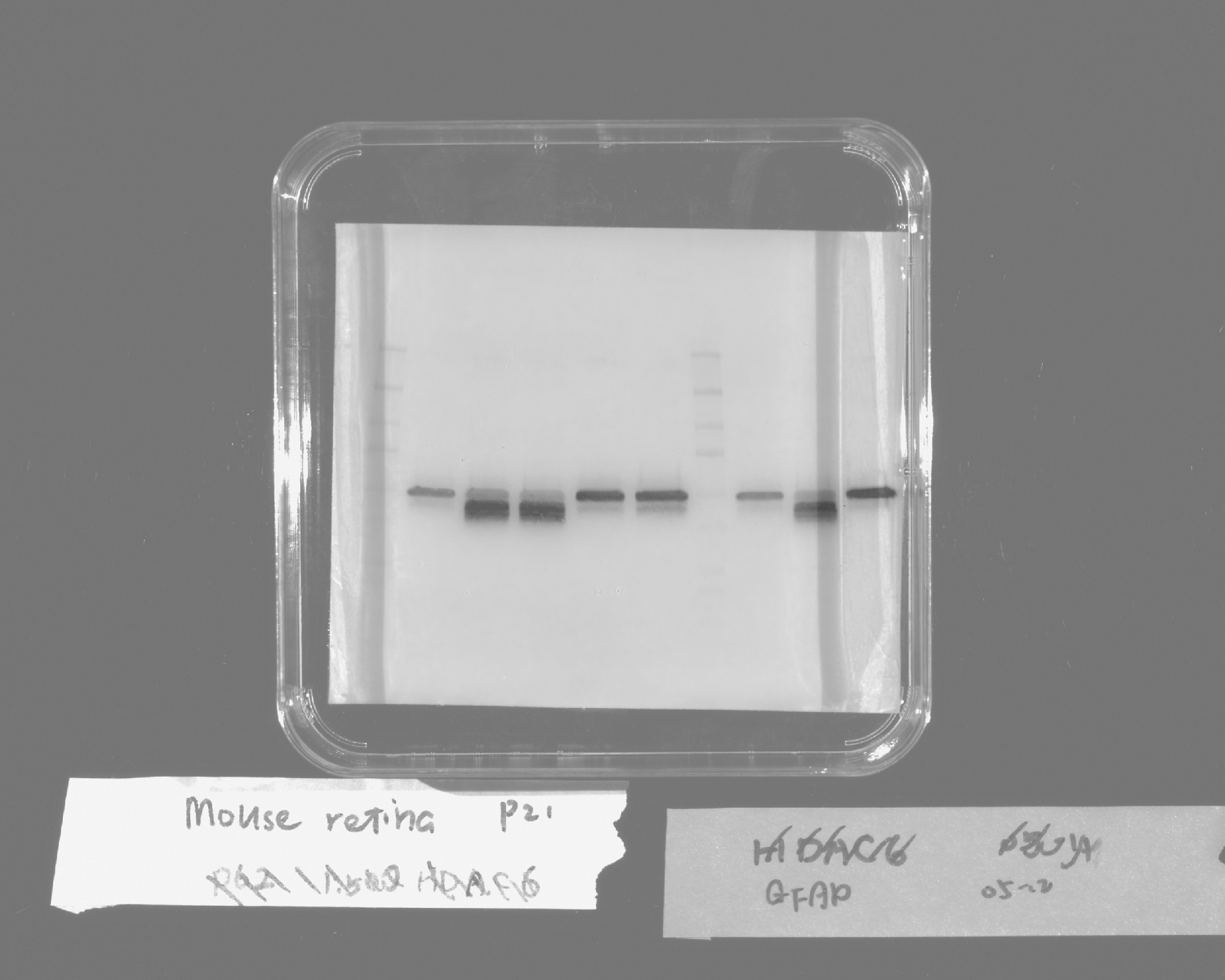

Supplement: Figure 6—figure supplement 3—source data 1. — The size of the protein ladders, Gfap, and relevant sample identity are labeled. [file elife-83205-fig6-figsupp3-data1.zip › Figure 6-figure supplement 3-source data 1/Figure 6-figure supplement 3-source data 1_Composite.tif]

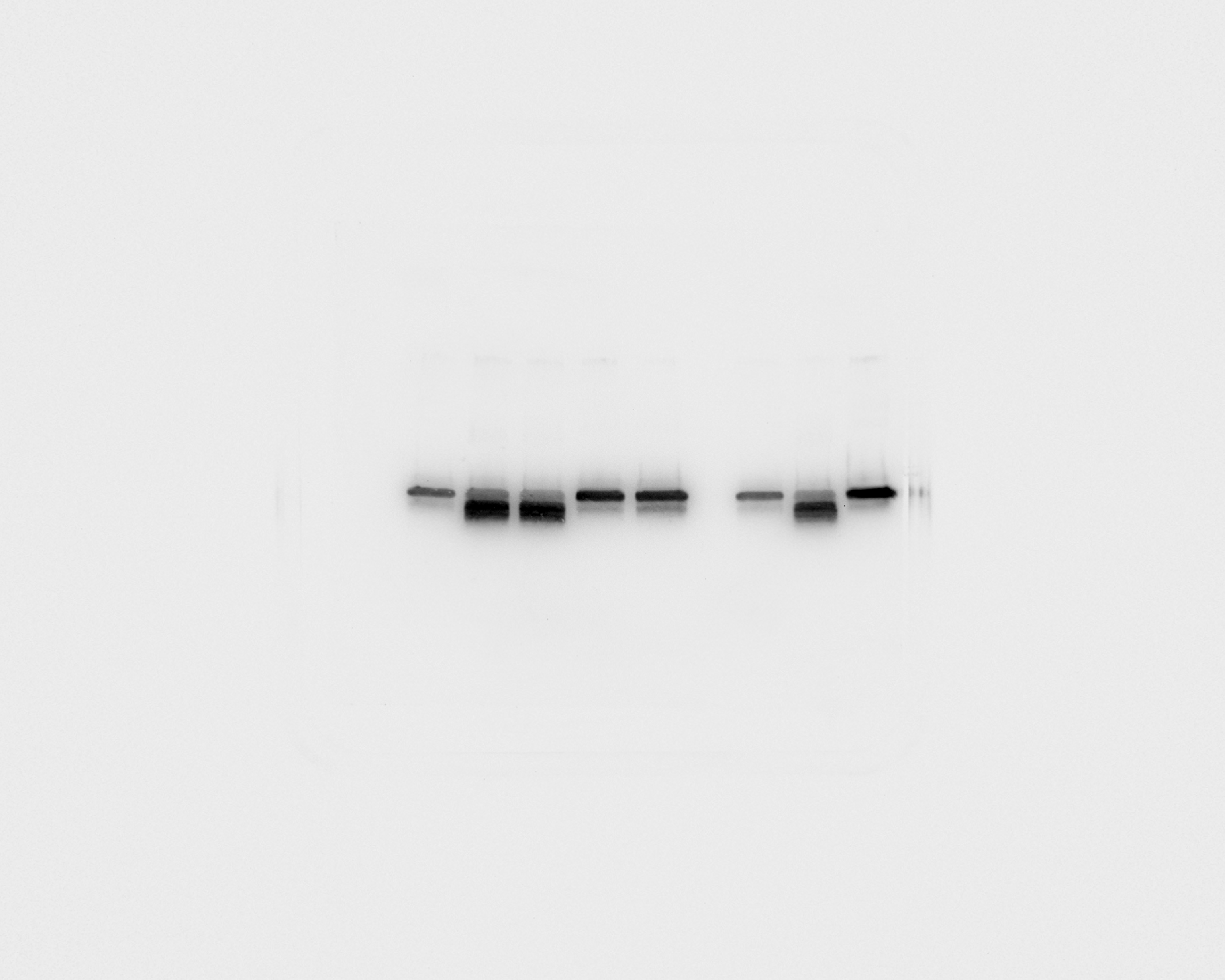

Supplement: Figure 6—figure supplement 3—source data 1. — The size of the protein ladders, Gfap, and relevant sample identity are labeled. [file elife-83205-fig6-figsupp3-data1.zip › Figure 6-figure supplement 3-source data 1/Figure 6-figure supplement 3-source data 1_Chemiluminescence.tif]

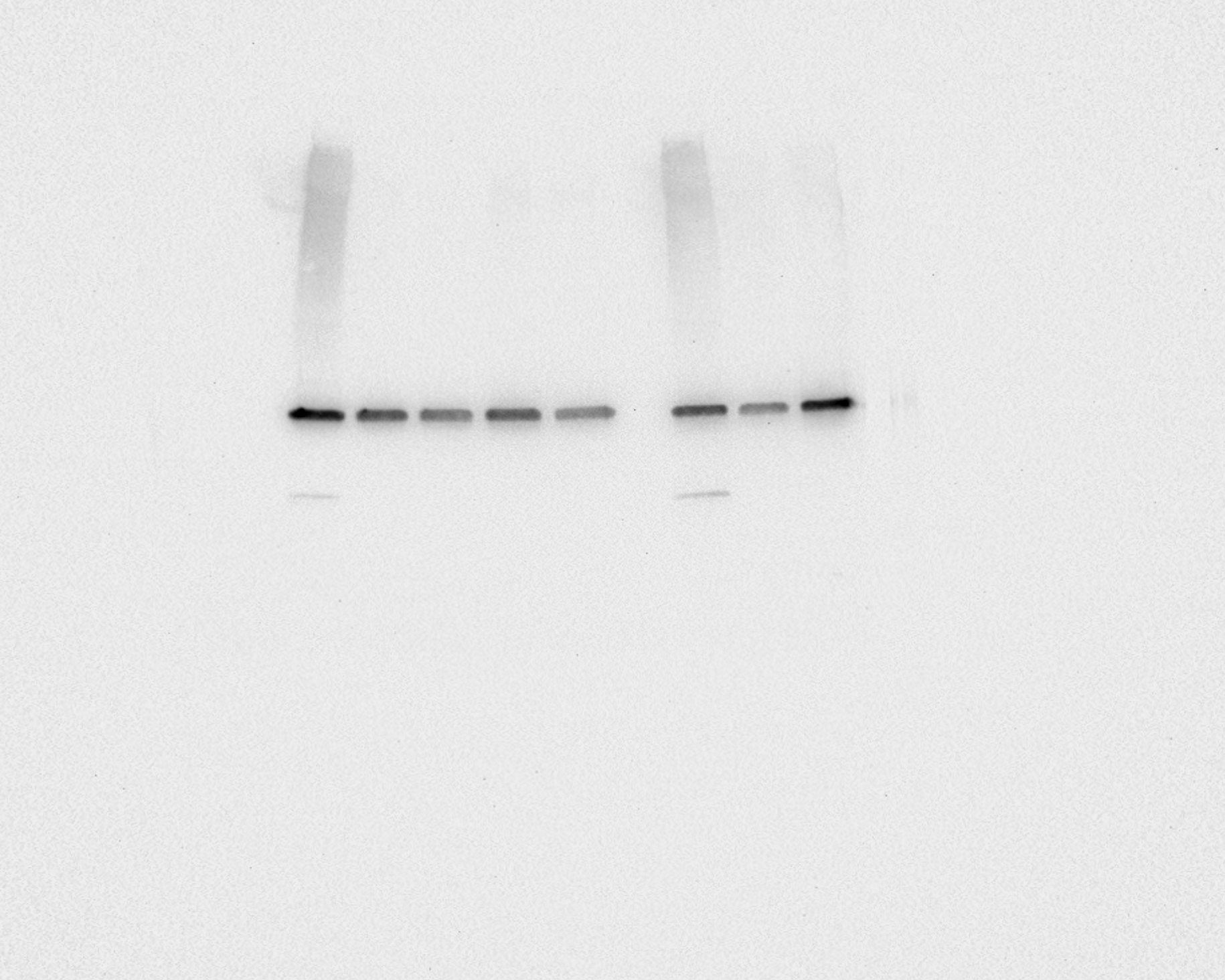

Supplement: Figure 6—figure supplement 3—source data 2. — The size of the protein ladders, g-Tubulin, and relevant sample identity are labeled. [file elife-83205-fig6-figsupp3-data2.zip › Figure 6-figure supplement 3-source data 2/Figure 6-figure supplement 3-source data 2_Chemiluminescent.tif]

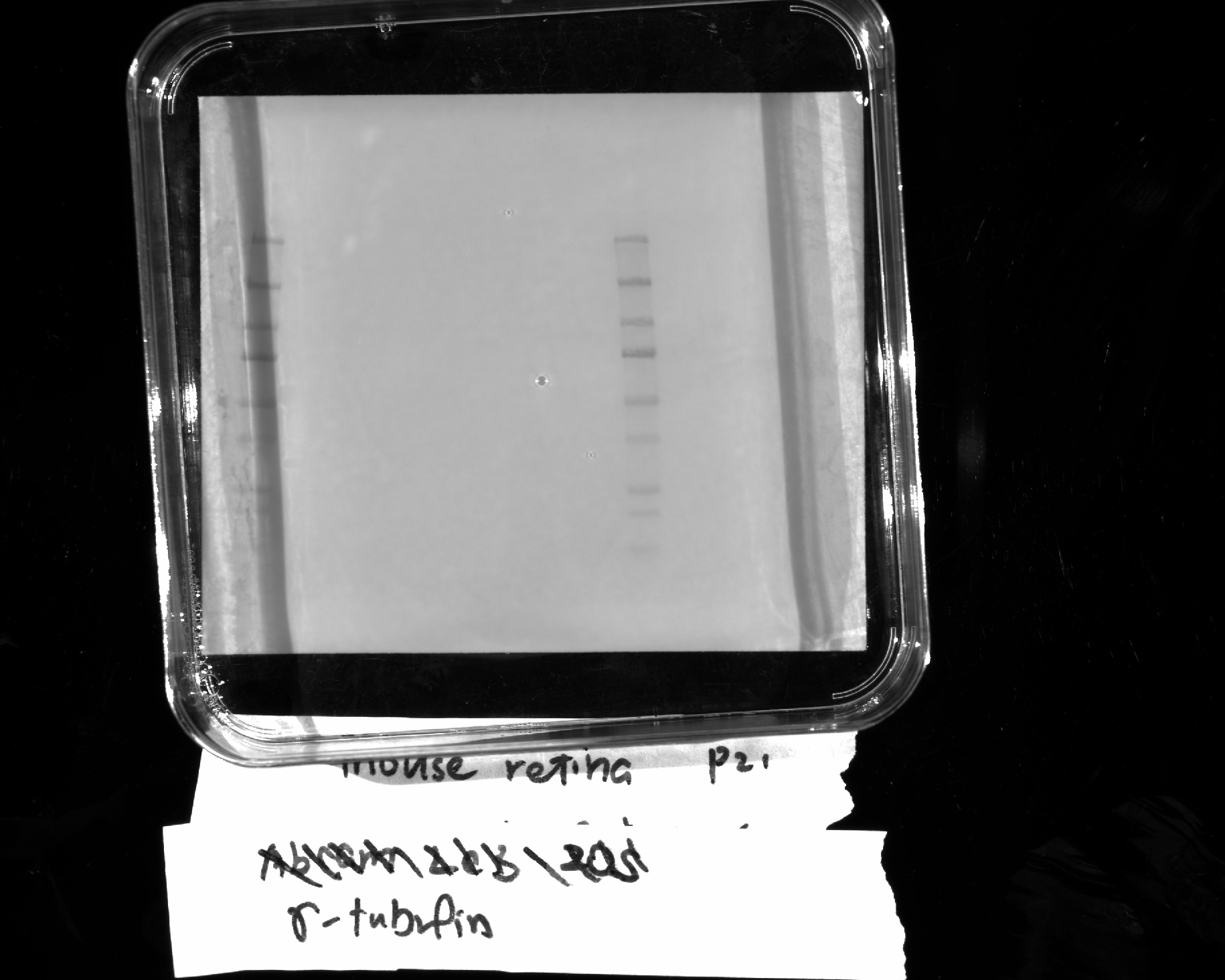

Supplement: Figure 6—figure supplement 3—source data 2. — The size of the protein ladders, g-Tubulin, and relevant sample identity are labeled. [file elife-83205-fig6-figsupp3-data2.zip › Figure 6-figure supplement 3-source data 2/Figure 6-figure supplement 3-source data 2_Colorimetric.tif]

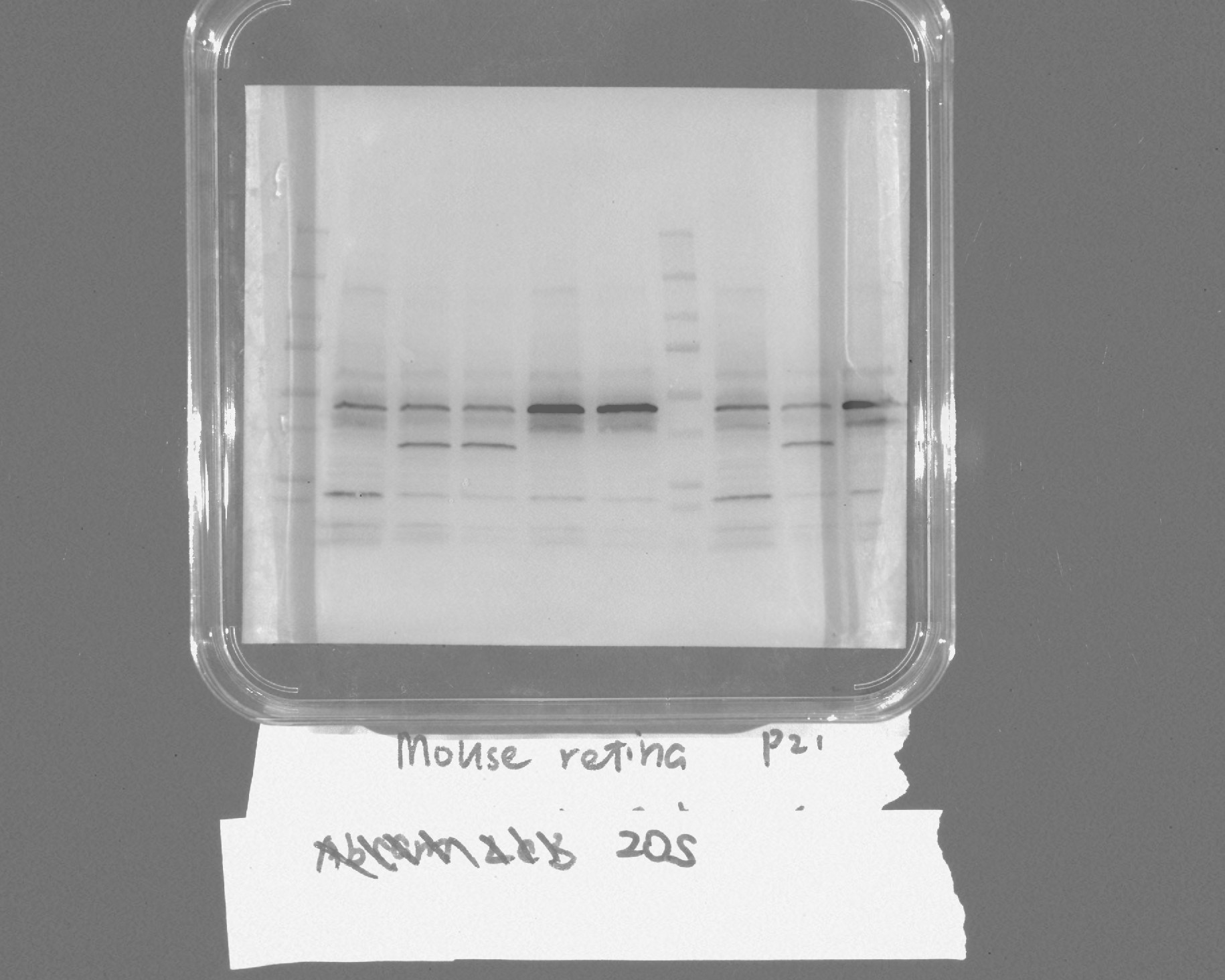

Supplement: Figure 7—source data 1. — WT and RSP stand for wild-type and reserpine respectively. The size of the protein ladders, 20 S proteosome, and relevant sample identity are labeled. [file elife-83205-fig7-data1.zip › Figure 7-source data 1/Figure 7-source data 1_Composite.tif]

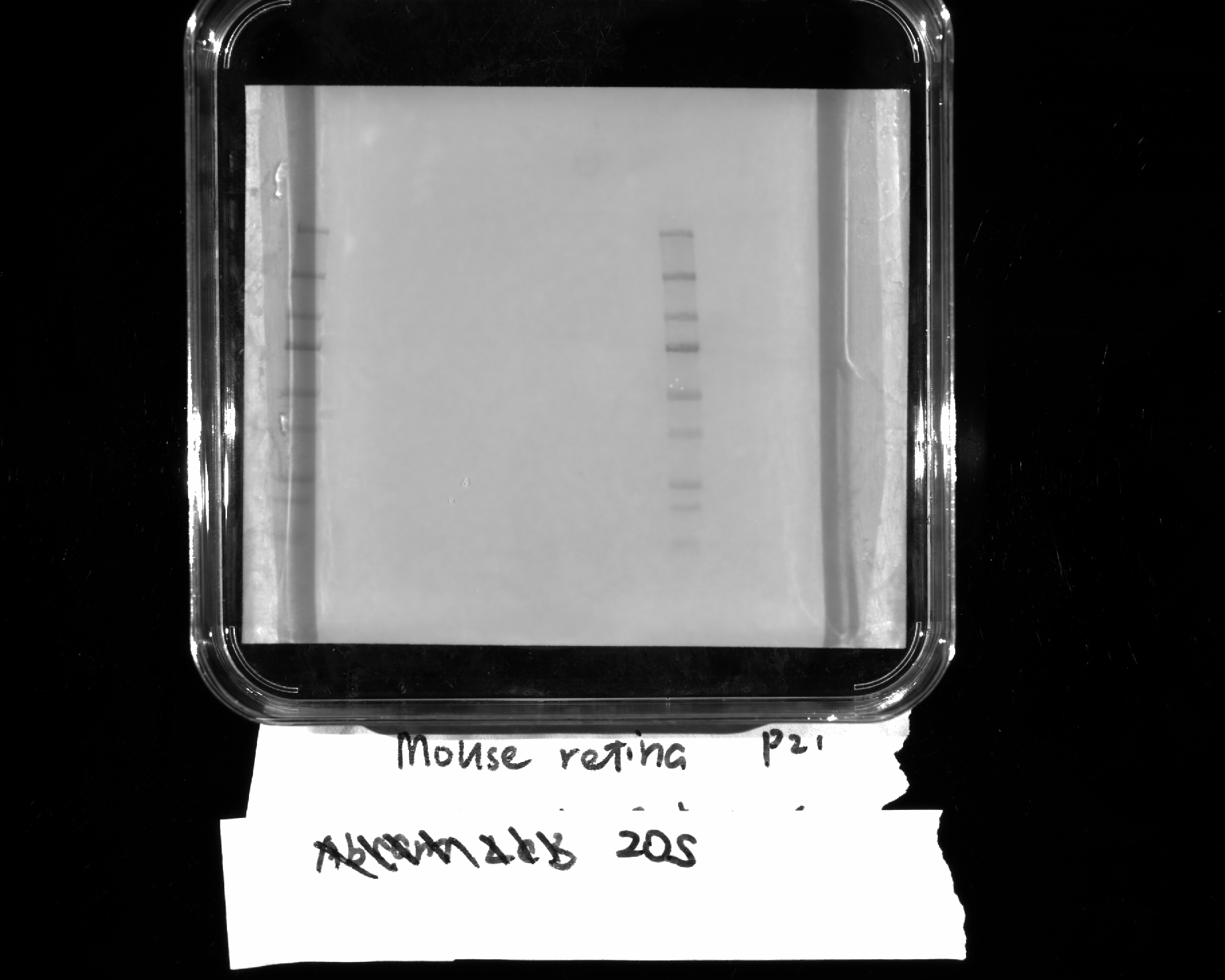

Supplement: Figure 7—source data 1. — WT and RSP stand for wild-type and reserpine respectively. The size of the protein ladders, 20 S proteosome, and relevant sample identity are labeled. [file elife-83205-fig7-data1.zip › Figure 7-source data 1/Figure 7-source data 1_Colorimetric.tif]

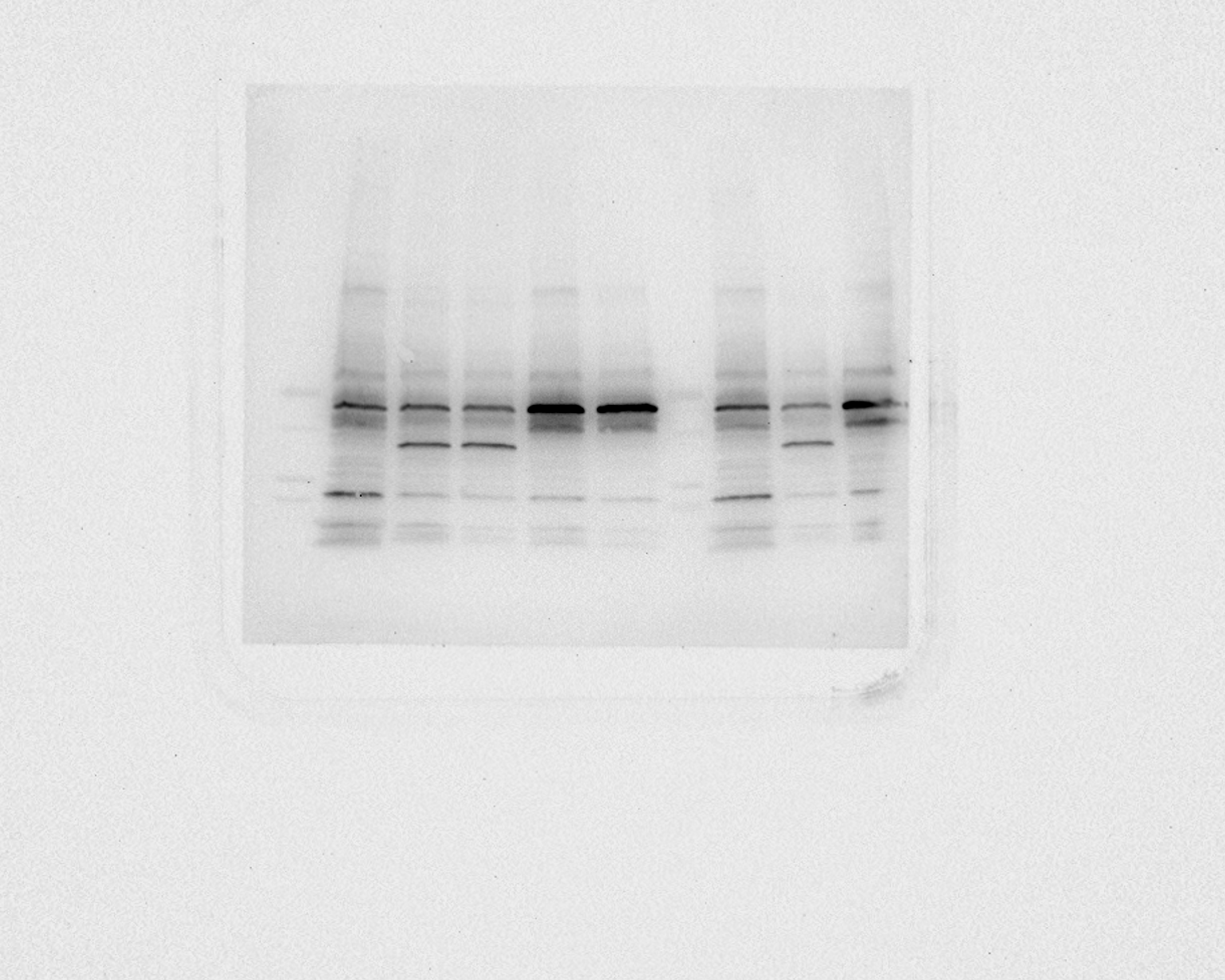

Supplement: Figure 7—source data 1. — WT and RSP stand for wild-type and reserpine respectively. The size of the protein ladders, 20 S proteosome, and relevant sample identity are labeled. [file elife-83205-fig7-data1.zip › Figure 7-source data 1/Figure 7-source data 1_Chemiluminescence.tif]

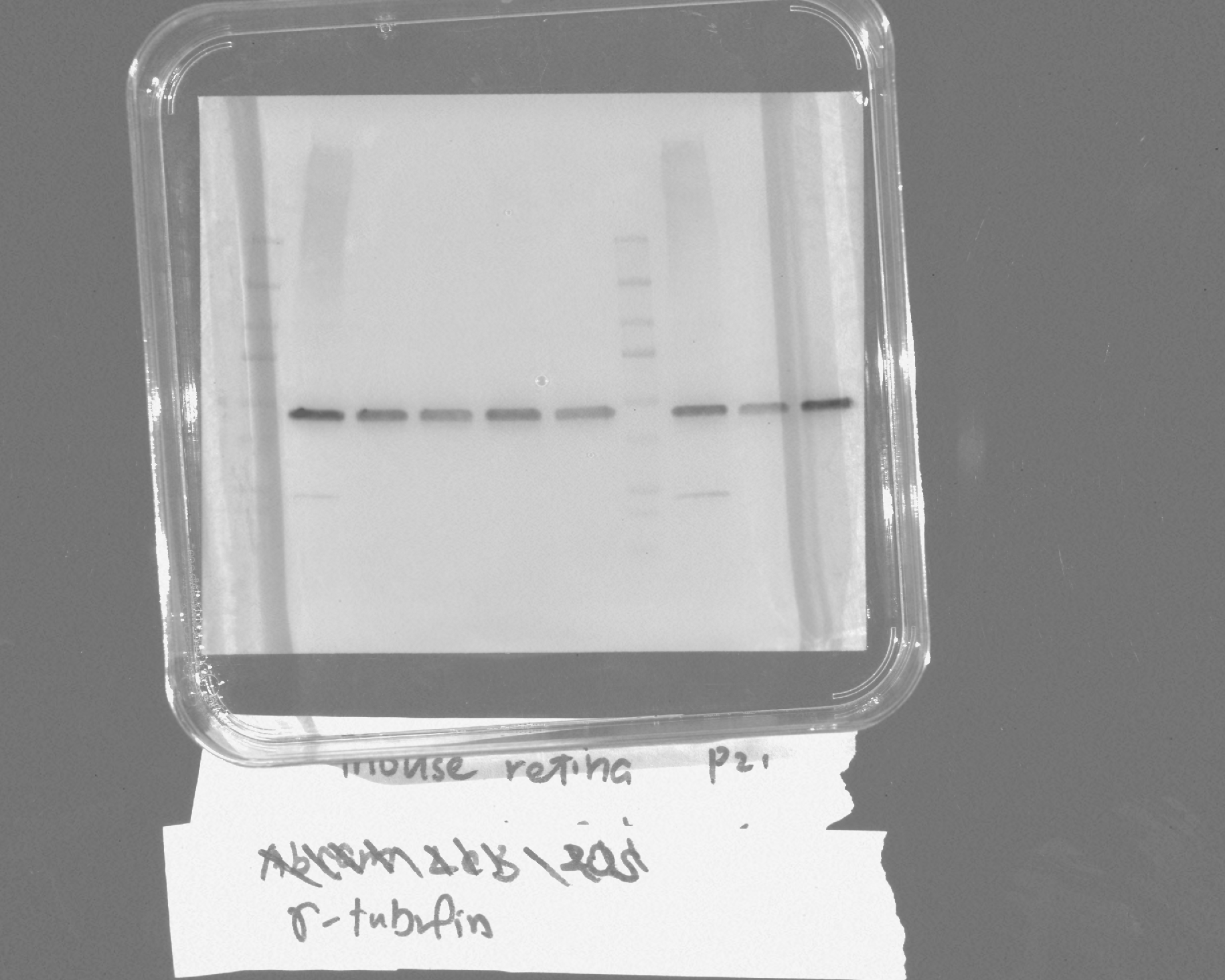

Supplement: Figure 7—source data 2. — WT and RSP stand for wild-type and reserpine respectively. The size of the protein ladders, γ-Tubulin, and relevant sample identity are labeled. [file elife-83205-fig7-data2.zip › Figure 7-source data 2/Figure 7-source data 2_Composite.tif]
